# Investigating mechanical and inflammatory pathological mechanisms in osteoarthritis using MSC-derived osteocyte-like cells in 3D

**DOI:** 10.3389/fendo.2024.1359052

**Published:** 2024-08-02

**Authors:** Sophie J. Gilbert, Ryan Jones, Ben J. Egan, Cleo Selina Bonnet, Sam L. Evans, Deborah J. Mason

**Affiliations:** ^1^ Biomechanics and Bioengineering Centre Versus Arthritis, School of Biosciences, Cardiff University, Cardiff, United Kingdom; ^2^ Biomechanics and Bioengineering Centre Versus Arthritis, School of Engineering, Cardiff University, Cardiff, United Kingdom

**Keywords:** osteocyte, mechanical load, osteoarthritis, inflammation, 3D model, RNAseq analysis

## Abstract

**Introduction:**

Changes to bone physiology play a central role in the development of osteoarthritis with the mechanosensing osteocyte releasing factors that drive disease progression. This study developed a humanised *in vitro* model to detect osteocyte responses to either interleukin-6, a driver of degeneration and bone remodelling in animal and human joint injury, or mechanical loading, to mimic osteoarthritis stimuli in joints.

**Methods:**

Human MSC cells (Y201) were differentiated in 3-dimensional type I collagen gels in osteogenic media and osteocyte phenotype assessed by RTqPCR and immunostaining. Gels were subjected to a single pathophysiological load or stimulated with interleukin-6 with unloaded or unstimulated cells as controls. RNA was extracted 1-hour post-load and assessed by RNAseq. Markers of pain, bone remodelling, and inflammation were quantified by RT-qPCR and ELISA.

**Results:**

Y201 cells embedded within 3D collagen gels assumed dendritic morphology and expressed mature osteocytes markers. Mechanical loading of the osteocyte model regulated 7564 genes (Padj p<0.05, 3026 down, 4538 up). 93% of the osteocyte transcriptome signature was expressed in the model with 38% of these genes mechanically regulated. Mechanically loaded osteocytes regulated 26% of gene ontology pathways linked to OA pain, 40% reflecting bone remodelling and 27% representing inflammation. Load regulated genes associated with osteopetrosis, osteoporosis and osteoarthritis. 42% of effector genes in a genome-wide association study meta-analysis were mechanically regulated by osteocytes with 10 genes representing potential druggable targets. Interleukin-6 stimulation of osteocytes at concentrations reported in human synovial fluids from patients with OA or following knee injury, regulated similar readouts to mechanical loading including markers of pain, bone remodelling, and inflammation.

**Discussion:**

We have developed a reproducible model of human osteocyte like cells that express >90% of the genes in the osteocyte transcriptome signature. Mechanical loading and inflammatory stimulation regulated genes and proteins implicated in osteoarthritis symptoms of pain as well as inflammation and degeneration underlying disease progression. Nearly half of the genes classified as ‘effectors’ in GWAS were mechanically regulated in this model. This model will be useful in identifying new mechanisms underlying bone and joint pathologies and testing drugs targeting those mechanisms.

## 1 Introduction

Abnormal joint loading through skeletal malalignment, age and obesity are key risk factors for osteoarthritis (OA) (1). At least 12% of the OA population are younger, with post-traumatic OA (PTOA) arising from prior joint injury (2), and although progression rate varies, injury can cause debilitating chronic pain and reduced mobility within 10 years. Changes to bone physiology play a central role in the development of OA (3) manifesting clinically as disruption of the tidemark, subchondral bone sclerosis and osteophyte formation alongside articular cartilage destruction. These changes are mediated by bone resorbing osteoclasts, bone forming osteoblasts, and osteocytes whose primary role is to maintain the integrity and function of bone (4) in response to its metabolic, mechanical and inflammatory environment, by regulating gene and protein expression. The osteocytes, which comprise 90–95% of all bone cells, release a variety of factors in response to mechanical load including nitric oxide (NO), prostaglandin E2 (PGE2), and receptor activator of nuclear factor kappa-B ligand (RANKL) to regulate osteoclast and osteoblast function as well as acting in an endocrine manner, releasing factors that target distant cells in other tissues (5–8). The extensive osteocytic network embedded within a lacuno-canalicular system throughout the bone, with cell-cell and cell-matrix connections that extend to the bone surface (9, 10) is ideally suited to the role of osteocytes as principal regulators of bone mechanosensation and mechanotransduction [reviewed in (11)].

In knee OA, mechanical loading causes pain, but the mechanism underlying the link between load and pain is unknown; bone is highly innervated and is one of the only tissues in the joint where structural changes correlate to pain in OA (12). Glutamate, the major excitatory neurotransmitter in the nervous system, also signals in peripheral and non-neuronal tissue. Both the regulation of glutamate release and the ability to respond to glutamate by expression of glutamate receptors (GluRs) has been reported in a range of joint cells including osteocytes (13–15). Glutamate signalling in bone is regulated by mechanical load (16) and linked to joint pain in humans (17). Increases in synovial fluid glutamate concentrations occur in both rheumatoid arthritis (RA) and OA patients (18) and correlate with an increase in inflammatory mediators in RA (19). In addition, synovial fluid glutamate levels in patients following anterior cruciate ligament (ACL) rupture and meniscal damage are comparable to that of OA patient synovial fluid levels and decrease with time post injury (13). Glutamate induces knee inflammation (20) and contributes to arthritic pain and swelling in an inflammatory arthritis model (21). Glutamate receptor antagonists within the joint alleviate symptoms of OA including pain, inflammation, and bone and cartilage pathology (13, 22). Inhibition of AMPA/KA GluRs with NBQX at the time of onset, improves pain related behaviour in rat inflammatory arthritis (antigen induced) and mouse PTOA model (ACL rupture) and reduces swelling and inflammation (13, 22). This protection is partly explained by NBQX inhibiting AMPA/KA GluR release of interleukin-6 (IL-6), an early driver of inflammation in both models of arthritis (15, 22, 23). IL-6 acts as a mechano-sensitive cytokine playing a key role in the biochemical control of bone remodelling (24, 25). Mechanical loading of osteocytes increases NO and IL-6 release and increases IL-6, osteoprotegerin (OPG), RANKL, and tumour necrosis factor-alpha (TNF-α) gene expression (26). Chronic IL-6 overexpression increases bone remodelling causing a net loss of bone by activating RANKL-induced bone resorption (27–29). Both pro-inflammatory and anti-inflammatory cytokines have been implicated in the pathogenesis of OA (30) with IL-6 among the most prominently elevated cytokine involved in the OA inflammatory response (31, 32). Increased circulating IL-6, as well as increased body mass index, predicts development of radiographic knee OA (33) and single nucleotide polymorphisms in the IL-6 gene are associated with radiographic hand OA (34). In addition, serum IL-6 levels are strongly associated with the incidence of age-related OA (35) and a predictive marker for the risk of OA progression (36). The role of IL-6 in OA pathophysiology has been studied in OA animal models showing that IL-6 is largely destructive (37) affecting both cartilage and subchondral bone (38).

Although mechanical load is the major driver of human OA, humanised *in vitro* osteocyte models investigating mechanics and its interactions with inflammation are very limited and have not been validated against *in vivo* models or human clinical data (39, 40). We have used our humanised *in vitro* model of osteocytes to detect early responses to either IL-6, a driver of degeneration and bone remodelling in animal (22, 37) and human (33, 34) joint injury, or mechanical loading, to mimic early OA stimuli in joints. Our osteocyte model (adapted from Vazquez et al. (41)) is derived from human Y201 stem cells differentiated into osteocytes in 3D type I collagen gels and mechanical strain applied using our custom loading device (adapted from Vazquez et al. (41)). The aim of this current study was to assess the effect of pathological mechanical load on the osteocyte signature and determine the influence of IL-6 on readouts that have been reported in OA. This will help identify mechanical and inflammatory mechanisms that cause pain or alter bone tissue structure *in vitro* and provide new mechanistic insight into disease progression.

## 2 Materials and methods

Chemicals were from Sigma (Poole, UK) and tissue culture and molecular biology reagents from Thermo Fisher Scientific (Invitrogen, Paisley, UK) unless otherwise stated and were of analytical grade or above. sIL6r and IL6 were from Peprotech, UK.

### 2.1 Cell culture

Y201 hTERT-MSCs, gifted from Prof Paul Genever (University of York), were used as a model human MSC line (42). These cells can rapidly differentiate in 3D to osteocytes in a manner similar to bone marrow MSCs in spheroid cultures (43) and maintain mechanoresponsive behaviour (44). Cells were cultured in basal medium [Dulbecco’s modified Eagle’s medium (DMEM), high glucose, pyruvate, GlutaMAX™, supplemented with 5% fetal bovine serum (FBS), 100 U/mL penicillin and 100 μg/mL streptomycin] at 37°C in a humidified atmosphere of 5% CO_2_, 95% air. At 80–85% confluency cells were sub-cultured by treating with TrypLE™. Y201 cells were incorporated into type I collagen gels at a concentration previously determined (41). Briefly, lyophilised rat tail tendon type I collagen was dissolved in 7mM glacial acetic acid and mixed 4:1 with 10X MEM containing 11g/L sodium bicarbonate on ice and neutralized [1M tris(hydroxymethyl)aminomethane (Tris) base, pH 11.5] to give 2mg/mL type I collagen gels. Y201 cells (0.125 x 10^6^ cells/gel or 0.05 x 10^6^ cells/gel for RNAseq) diluted in αMEM (<10% of total gel volume) were added to the collagen on ice and 250μL distributed into 48-well plastic (phenotype studies and IL-6 studies) or silicone (loading studies (41)) plates (**Supplementary Methods 1**), for polymerization at 37°C. After 1 hour, 800μL basal medium supplemented with osteogenic differentiation factors (50µg/mL ascorbate-2-phosphate, 5mM β-glycerophosphate, 1nM dexamethasone) was added onto the surface of the gels and cells cultured at 37°C with media changes every 3–4 days for the indicated periods.

#### 2.1.1 Assessment of cell viability

Cultures grown in plastic plates for 7 days were rinsed with phosphate buffered saline, pH 7.3 (PBS), incubated with 1µL hoescht (1mg/mL) and 4µL propidium iodide (100µg/mL) in serum free medium for 2 hours at 4°C and then for a further 2.5 hours at 37°C before washing overnight at 37°C in normal culture medium with gentle agitation. Cells were fixed in 1% (wt/vol) paraformaldehyde for 30 minutes at 4°C, washed in PBS prior to overnight infiltration with 50% OCT compound (Tissue Tek) in PBS at 4°C. Gels were frozen in fresh OCT compound onto cryostat stubs using dry ice and cryosections cut at 20μm using a Bright OTF5000 cryostat and collected on polysine slides (VWR, Lutterworth, UK). Slides containing sections were mounted in VECTASHIELD^®^ Mounting Medium containing DAPI (1.5 μg/mL) to counterstain DNA (Vector Laboratories, Peterborough, UK) and viewed using a light microscope (BX61, Olympus).

#### 2.1.2 Assessment of cell phenotype

##### 2.1.2.1 Cell shape and immunolocalization of osteocyte markers

Cultures grown in plastic plates for 7 days were rinsed with PBS, and gels fixed in 1% (wt/vol) paraformaldehyde, frozen and sectioned as outlined above. Sections were stained with phalloidin to assess cell morphology or processed for immunocytochemistry. Phalloidin-iFluor conjugate staining was carried out according to manufacturer’s protocol (Abcam). Slides were removed from -20°C, allowed to equilibrate to room temperature and hydrated with 2mL of PBS per section. PBS was aspirated off, sections permeabilised with 0.1% Triton X-100 in PBS for 5 minutes and washed 3x with PBS prior to treatment with 100μL of 1X phalloidin conjugate (1μL per 1mL of PBS + 1% BSA) per section. Sections were left to stain for 60 minutes at room temperature whilst protected from light and washed 3x with an excess volume of PBS prior to mounting in VECTASHIELD^®^ Mounting Medium containing DAPI (1.5 μg/mL). Stained sections were visualised using fluorescent imaging (BX61, Olympus). For immunocytochemistry, each step was performed at room temperature unless stated otherwise and between each incubation step, sections were washed 3x 5 min in 0.01 M PBS containing 0.001% Tween 20 (wash buffer). All antibodies were diluted in wash buffer. Cells were washed before blocking in 2% (v/v) normal goat serum (Dako UK, Ely, UK) for 1 hour. After overnight incubation at 4°C with a rabbit polyclonal primary antibody to Sclerostin (Abcam; ab75914) diluted 1:100, cells were washed before incubating for 1 hour with goat anti-rabbit Alexa 488 conjugated secondary antibody (4 μg/mL; Molecular probes, Invitrogen). Finally, after washing, cells were mounted in VECTASHIELD^®^ Mounting Medium containing DAPI (1.5 μg/mL). Representative cells from multiple fields of view were imaged by confocal microscopy (Leica TCSSP2, Germany) using a 63x oil immersion objective lens and appropriate settings for AlexaFluor 488 (green) and DAPI (blue). Negative controls where the primary antibody was omitted or replaced with rabbit IgG were devoid of fluorescent signal (data not shown).

##### 2.1.2.2 RT-PCR analysis of osteocyte markers

RNA was extracted from cells grown in gels from duplicate cultures at day 7 and analysed for the expression of osteocyte markers by RT-qPCR. Briefly, gels were placed into 600µl buffer RLT Plus (RNeasy Plus kit, Qiagen) and 6µl β-mercaptoethanol added prior to loading onto a QIAshredder spin column to disrupt the gels. Samples were centrifuged for 2 minutes at full speed and the lysate added to gDNA Eliminator spin columns to digest genomic DNA. Samples were processed according to manufacturer’s instructions (Qiagen) and RNA eluted in 30µl RNAse/DNAse free water. RNA quality and concentration were assessed by TapeStation Analysis (Agilent Technologies, UK). cDNA was generated in a 20 µL reaction from 500 ng RNA using 50 ng random hexamers (0.5 mg/mL; Promega) RNasin® RNase Inhibitor (40U), 5mM DTT, 0.5mM each dNTP, and Superscript IV reverse transcriptase (200 units). Gene expression was measured by SYBR green RT-PCR using the Platinum™ SYBR™ Green qPCR mix and the AriaMX qPCR system according to manufacturer’s instructions (Agilent Technologies UK) with 200 nM forward and reverse primers (**Supplementary Table 1A**) and the following cycle conditions: 1 cycle of 95°C, 3 minutes; 40 cycles of 95°C, 15 seconds and 60°C, 30 seconds; 1 melt cycle of 95°C, 1 minute, 65°C 30 seconds, 95°C 1 minute.

### 2.2 Mechanical loading of collagen gels

#### 2.2.1 Strain validation in collagen gels

Force strain relationships were validated in collagen gels within the custom-built Cardiff loading device which comprises a deformable silicone multiwell plate within a 3D printed loading device (**Supplementary Figures 1A, B**). Defined vertical displacements applied with a Bose Electroforce 3200 machine (TE Instruments) extend the levers outwards and caused the plate to stretch. Collagen gels (2mg/mL) containing 500μL of blue- and violet-coloured microspheres (10μm, Polysciences, Park Scientific Ltd, UK) subjected to vertical displacements of 0- 2.1mm at 0.35mm intervals were imaged by light microscopy and a tracking code, written in Matlab calculated the strain/displacements relationships (**Supplementary Figures 1C–E**). There was a linear relationship between displacement and strain up to displacements of 0.7mm. A displacement of 0.7mm represented a pathophysiological load of 4300µϵ ( ± 103) and was used for all experiments.

#### 2.2.2 Mechanical loading

For loading, 3D Y201 cultures were prepared and cultured in the silicone plate in 800μL of basal medium containing osteogenic differentiation factors and incubated at 37°C in 5% CO2/95% air atmosphere for 5-days. After this time, the media were replenished and left for 24 hours. One hour prior to loading, media were removed and 800μL osteogenic media added. An hour later, silicone plates were loaded using a BOSE ElectroForce^®^ 3200 loading instrument (TE Instruments, UK) to stretch the plate causing cyclic compression in all wells (pathophysiological load 4300μϵ induced by 0.7mm displacement, 10Hz, 3000 cycles (41, 45, 46). The loading regime was chosen to recapitulate *in vivo* models where validated high physiological strains induced osteogenesis (45, 47, 48). This high (pathophysiological) strain down regulated sclerostin (**Supplementary Figure 1F**) a known osteocyte derived mechanoresponsive molecule that is a potent regulator of bone formation (46) as well as inflammatory mediators relevant to osteoarthritis (41). In addition, osteocytes respond to mechanical load within seconds, revealing gene expression changes within 1 hour (49) and protein changes 24–72 hours later (41). Control gels in the silicone plate were placed into the loading device but received no load. Loading was controlled using WinTest^®^ Software 4.1 with TuneIQ control optimization (BOSE). Media was collected after 1hr and 24hrs, aliquoted and frozen (-20°C) for analysis of released factors. Gels, 1hr post load, were placed into 600µL buffer RLT Plus (RNeasy Plus kit, Qiagen) and stored at -80°C prior to RNA extraction.

#### 2.2.3 Confirmation of the osteocyte response to load

RNA was extracted 1 hour post load using RNeasy Plus kits, RNA eluted in 30µl RNAse/DNAse free water, cDNA synthesised and RTqPCR performed as described above (section 2.1.2.1). Reference genes, 36B4, YWHAZ, RPL13A, 18S, β-actin, GAPDH were tested across experimental conditions. The geometric mean of YWHAZ and 18S (stability value 0.292), were identified by RefFinder (50) as the most stable and used to calculate fold change relative to untreated cells using the ΔΔCT method (51). All primers (**Supplementary Table 1A**) were purchased from MWG and validated using a standard curve of five serial cDNA dilutions with primer efficiencies between 90–110% (52).

#### 2.2.4 RNAseq analysis: generation of the osteocyte ‘mechanosome’

RNA was extracted from loaded samples 1 hour post load (n=6) and unloaded controls (n=5) using RNeasy Plus kits as described above (section 2.1.2.2). RNA was eluted in 30µl RNAse/DNAse free water and RNA quality, and concentration assessed by TapeStation Analysis (Agilent). An RNA sequencing library was prepared for the mechanically loaded and control samples, using the New England Biolabs Ultra II directional RNA library prep kit (Wales Gene Park). cDNA was synthesized using this RNA which, after undergoing fragmentation, had adaptors ligated to the ends. The MiSeq Nano system (Illumina) was used to complete a sequencing library quality control after which sequencing was performed using the NovaSeq 6000 system (Illumina) running a 2 x 100bp paired-end reads run on a NovaSeq S1 flow cell. Trimming to remove adapter sequencer and poor-quality ends of reads was performed by Trim Galore using default parameters in paired-end mode. Trimmed paired-end reads were aligned to the GRCh38 no_alt_plus_hs38d1 analysis set reference using STAR (v2.5.1b), an ultrafast universal RNAseq aligner, following the 2-pass method (53). QC metrics were generated using FastQC (v0.11.2), and summary statistics were generated using Samtools (v0.1.19) flagstat. Raw counts were calculated for all samples for both (i) exons and (ii) genes using Subread featureCounts Version 1.5.1. Counts were generated for paired end read fragments summarized at exon level and then aggregated at transcript level. Ambiguity between the labels on sample C1 and L1 and the suspicion that these 2 samples had been inadvertently switched led to an additional PCA analysis which showed C1 to cluster with loaded samples (**Supplementary Figure 2**); because of this C1 and L1 were excluded from further analysis leaving n=4 controls and n=5 loaded samples. Differentially expressed genes were identified using an DEseq2 analysis (54) on normalised count data. The resultant p-values were corrected for multiple testing and false discovery issues using the FDR method (55). RNAseq data are available from Mendeley Data (DOI: 10.17632/5md5rnybcs.1).

#### 2.2.5 Human osteoprotegerin and glutamate ELISAs

Media collected 1 hour, and 24 hours post-load were analysed for the release of Glutamate (KA1909, Bio-Techne) and OPG (AB100617, Abcam) using commercial kits following manufacturer’s instructions.

#### 2.2.6 Cytokine analysis

Frozen media samples were thawed on ice and centrifuged for 5 minutes at 3000 rpm, and 23 cytokines profiled (**Supplementary Table 1B**) in each sample by Luminex bead based multiplex assay using a Merck Milliplex^®^ MAP human cytokine/chemokine magnetic bead panel kit (2923824 HCYTOMAG-60K-23) following the manufacturer’s instructions.

### 2.3 sIL6r/IL6 treatment of Y201 cells in 3D gels

Y201 cells were embedded in 3D type I collagen gels as described above at a density of 0.125 x 10^6^ cells/gel and grown in 48-well plastic plates for 24 hours in basal media at 37°C, 5% CO2. Media was replaced with 800μL osteogenic media and cells cultured for 7 days with media changes every 3 days. At day 7, the media was replaced with 800μL osteogenic media containing IL-6 (5ng/mL) and sIL-6r (40ng/mL) in all but the control wells (26, 31, 32). At 24 and 72 hours, media was removed and stored at -20°C for cytokine analysis. TRIzol™ reagent (500μL) was added to each gel at 24 hours and pipetted repeatedly to dissolve the gels and lyse the cells, prior to storage at -80°C.

#### 2.3.1 RNA extraction and RT-qPCR analysis of gene expression

Total RNA was extracted from gels using TRIzol™ reagent according to the manufacturer’s protocol. RNA was DNase treated to remove genomic DNA (Ambion; Applied Biosystems, UK) and re-suspended in 50 µL RNase-free water. RNA integrity and concentration were assessed by Nanodrop™. cDNA was generated in a 20 µL reaction from 150 ng RNA as described above (section 2.1.2.2). RT-qPCR was carried out as described above using the geometric mean of EEF and RPL13A (RefFinder stability value 0.375) for normalisation.

#### 2.3.2 Cytokine analysis

Aliquoted media samples were centrifuged to remove cells and supernatants vortexed briefly prior to use. A multiplex electrochemiluminescence (ECL) kit (**Supplementary Table 1C**; U-Plex Proinflam Combo 1 Human K15049; Meso Scale Discovery, USA) and single-plex ELISAs (Glutamate KA 1909 Abnova, OPG RDR-OPG-Hu 2bScientific) were utilised to measure levels of released molecules according to manufacturer’s instructions. Multiplex ECLs were carried out in the Central Biotechnological Services (Cardiff University) utilising a Mesoscale discovery (MSD) plate reader to determine chemiluminescence measurements.

### 2.4 Statistics and data analysis

Results are presented as mean ± SEM. Graphs show individual data points, box and whisker plots of minimum and maximum values, 25th and 75th quartiles and median. Data were tested for normality and equal variances prior to transformations where necessary and appropriate statistical testing as indicated in the figure legends (Minitab 20). Differences were considered significant at *p=0.05*. For all statistics, unless stated otherwise, treatments were compared to untreated controls. Functional gene enrichment and pathway enrichment analysis were performed on up and down DEG sets using gProfiler (56) and Enrichr databases (57–59), respectively. RNAseq data was compared to the osteocyte signature (60) as well as a list of gene ontology terms (GO terms), compiled using AmiGO (61–63) and the search terms ‘bone’, ‘pain’, ‘inflammation’, and ‘mechanical load’. For phenotype studies, data is collected from 4 independent studies (N=4). For IL-6 effects on bone remodelling, OPG gene data is representative of 3 independent studies (N=3).

## 3 Results

### 3.1 Osteocyte–like cell viability and phenotype

**Figure 1:**
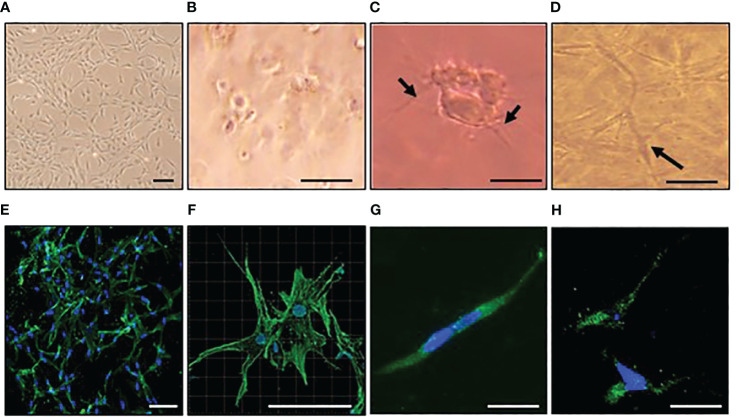
Osteocyte–like cells differentiated from Y201 mesenchymal stem cells are viable with an appropriate phenotype. Y201 mesenchymal stem cells were expanded in culture **(A)** prior to embedding in type I collagen gels **(B-D)**. Within 1 hour of culture **(B)**, cells were beginning to send out dendritic processes which were evident by 4 hours **(C)**. A 3D network of interconnecting cells was clearly evident by day 7 **(D)**. Phalloidin staining of cells revealed dendritic processes **(E, F)**; green) and sclerostin expression **(G, H)**; green) at day 7 of culture. DAPI nucleus = blue. Images were captured using a x4 **(A)**, x10 **(E)**, x20 **(B-D, G, H)**, and x40 **(F)** objective. Scale bar = 20µm B-D, G,H; 100 µm **(A, E, F)**.

Y201 cells produced dendritic processes within one hour of embedding in 3D collagen gels and formed interconnected networks by day 7, remaining viable (data not shown) and osteocyte-like (**Figures 1A–E**). After 7 days in 3D collagen gels, phalloidin staining revealed a dendritic morphology (**Figures 1E, F**; **Supplementary Video 1**) and immunolocalization confirmed sclerostin protein expression (**Figures 1G, H**). Cells did not proliferate between days 5 and 7 (data not shown). RTqPCR analysis confirmed cells expressed osteocyte markers sclerostin (SOST) and podoplanin (PDPN) and expressed osteocalcin (BGLAP), osteoprotegerin (TNFRSF11B), and type I collagen (COL1A1) (**Supplementary Figure 3A**).

### 3.2 Differentially expressed genes, model reproducibility and the osteocyte signature

**Figure 2:**
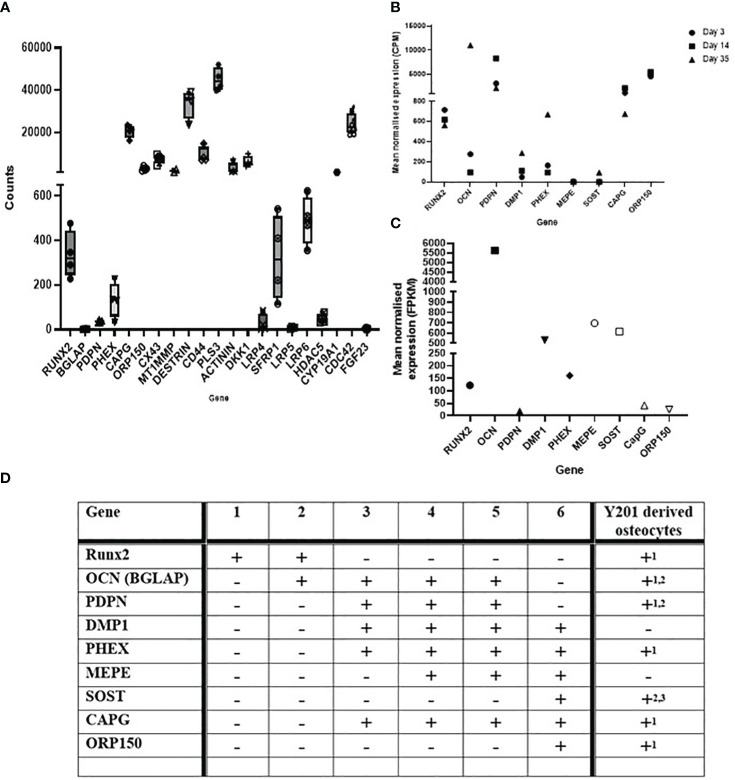
Osteocyte–like cells differentiated from Y201 mesenchymal stem cells express phenotypic markers of osteocyte development and maturation. RNAseq data quantifying gene expression associated with the development and maturation of osteocytes and the mineralisation process in **(A)** the Y201 osteocyte model used to generate the mechanosome. Data is compared to **(B)** IDGSW3 cells at day 3, 14 and 35 of differentiation (60), and **(C)** osteocyte-isolated samples from the Osteocyte Enrichment cohort (64). B and C were plotted using data publicly available (60, 64), respectively. **(D)** table [adapted from (10)] showing the relative temporal expression of various osteogenic markers during the transition from osteoblast to osteocyte where 1 = preosteoblast; 2 = osteoblast; 3 = embedding osteoblast; 4 = osteoid osteocyte; 5 = mineralizing osteocyte; 6 = mature osteocytes compared to expression of these markers in the Y201 derived osteocytes (1 = mechanosome RNAseq data, 2 = RTqPCR data, 3 = immunohistochemistry, detected = +, not detected = -).

RNA extracted from cells differentiated in silicone plates 1 hour post load was of high quality (RIN scores >9; **Supplementary Figures 3B**). RT-qPCR analysis revealed SOST expression was decreased by loading thereby confirming that the model cells were responding appropriately to load (**Supplementary Figure 1F**). RNA was processed for RNAseq and the dataset generated analysed for the presence of genes involved in the development and maturation of osteocytes and the mineralisation process (**Figure 2A**). Our data was compared to publicly available data generated from IDGSW3 cells at day 3, 14 and 35 of differentiation (**Figure 2B**) (57), and osteocyte-isolated samples from the Osteocyte Enrichment cohort (**Figure 2C**) (65) and the relative temporal expression of various osteogenic markers during the transition from osteoblast to osteocyte summarized [**Figure 2D**; adapted from (10)].

Hierarchical clustering of the sample set grouped replicates and separated biological conditions (**Supplementary Figure 2C**). Principal component analysis (PCA) characterised further the experimental variability revealing loaded samples L2 and L3 to be different to L4-L6 (**Supplementary Figures 2D, E**).

In total 7564 genes were differentially regulated (Padj p<0.05): 3026 down and 4538 up regulated by mechanical load (**Supplementary Table 2A**). Of these, 3824 genes were up regulated and 532 down regulated fold change (FC) >2. A volcano plot representing the log of the adjusted P value as a function of the log ratio of differential expression shows differentially regulated genes as red dots or triangles, the latter corresponding to genes where log2 FC is too low/high to be displayed on the plot (**Supplementary Figure 2F**). We compared our *in vitro* osteocyte ‘mechanosome’ data to the 1004 protein encoding genes from the published osteocyte signature representing human genes enriched in osteocytes relative to bone marrow and other osteoblast lineage cells (60). This revealed that 937/1004 osteocyte signature genes were expressed (**Supplementary Table 2B**) and 67 were not expressed in our dataset (**Supplementary Table 2C**). Of these, 379 were regulated by mechanical load (248 UP, 131 DOWN; Padj<0.05; **Supplementary Table 2D**). Functional gene enrichment analysis of the DEGs using gProfiler revealed alterations in genes involved in several biological processes, molecular function, cell compartments, and the reactome (**Supplementary Table 3A**). Analysis of the up regulated gene set revealed enrichment of genes including those involved in metabolic processes, cell response to stress, and cell component organization as well as genes involved in post-translational modification and toll-like receptor signalling (**Supplementary Table 3A** GEA.UP). Down regulated genes were enriched in RNA processes, and cilium processes (**Supplementary Table 3A** GEA.DOWN). Additional gene ontology searches were performed for terms related to mechanical load including ‘response to mechanical signalling’, ‘mechanically gated ion channels’, ‘mechanosensory behaviour’, ‘cell response to mechanical stimulus’, and ‘detection of mechanical stimulus’ revealing several regulated genes (**Supplementary Table 3B**). Of the 166 genes listed across these terms, 46 genes were upregulated and 24 downregulated by mechanical load (padj <0.05).

### 3.3 Mechanical load of osteocytes regulates readouts of osteoarthritis

Gene ontology searches were performed for terms related to readouts of OA including pain (**Supplementary Table 3C**), bone remodelling (**Supplementary Table 3D**) and inflammation (**Supplementary Table 3E**).

#### 3.3.1 Pain and the glutamate signalling pathway

Pathophysiological loading of osteocytes in our 3D model regulated genes known to be involved in gene ontology pathways linked to OA pain including genes related to sensory perception of pain and neuropathic pain and members of the glutamate signalling pathway (**Supplementary Table 3C**). Of the 253 genes listed across these terms, 43 genes were up regulated, and 22 genes downregulated by mechanical load (Padj < 0.05, **Supplementary Table 2A**). GRIK1, GRIA4, GRIN2B, GRIN2C, GRIN2D, GRIN3A, SLC1A2, SLC1A3, GRID2 were expressed but not regulated by load (data not shown). In addition, COL12A1 and COL16A1, present in bone marrow lesions (BMLs) (66) which correlate to OA pain (67), were upregulated 4- (padj=0.000) and 1.7-fold (padj= 0.049) by load, respectively (data not shown).

**Figure 3:**
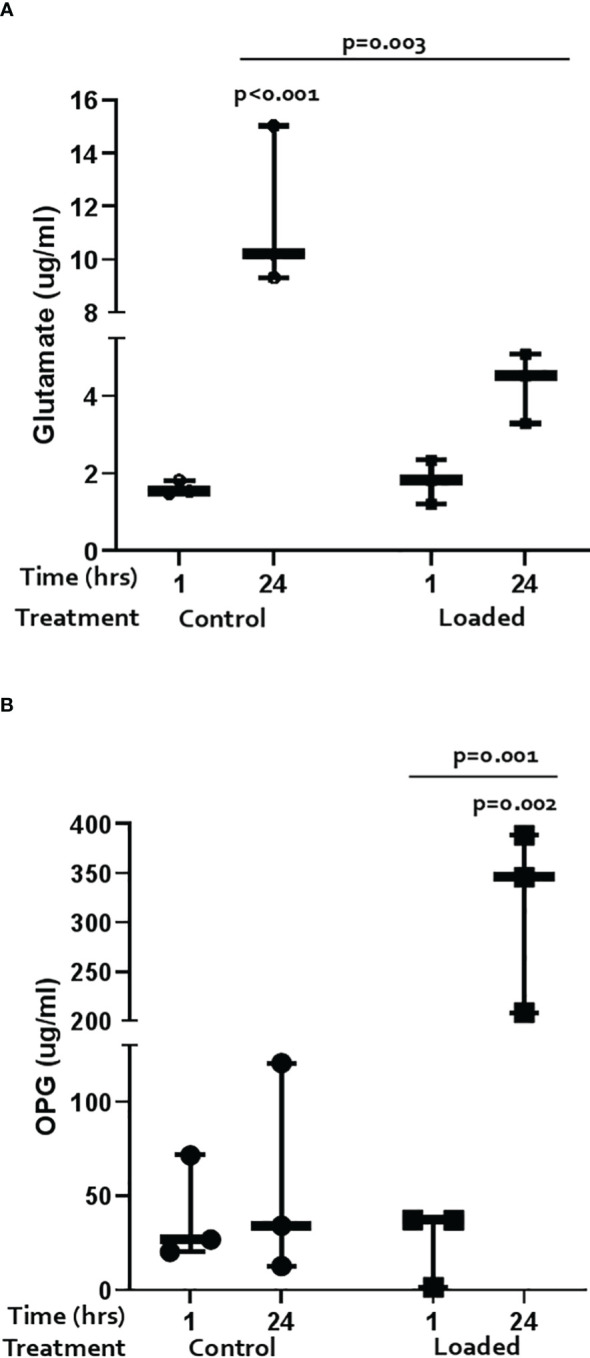
Regulation of markers of pain and bone remodelling markers in the 3D osteocyte model. The amount of **(A)** glutamate and **(B)** OPG released into the media from loaded osteocytes at 1 hour and 24 hours post-load (n=3/treatment, GLM ANOVA and Tukey’s post-hoc tests).

Glutamate release was measured by ELISA (**Figure 3A**). Over the 24-hrs of culture, cells increased release of glutamate into the media; loading dampened this response (control 1-hr vs 24-hr 7-fold, p<0.001; loaded 2.7-fold, p=0.003).

#### 3.3.2 Bone remodelling

Pathophysiological loading of osteocytes in our 3D model regulated genes known to be involved in gene ontology pathways linked to bone remodelling including ossification, bone resorption, and bone mineralisation (**Supplementary Table 3D**). Of the 217 genes listed across these terms, 49 genes were upregulated and 31 downregulated by mechanical load (Padj < 0.05). In addition, RANKL (TNFSF11) and OPG (TNFRSF11B) were expressed but not regulated by mechanical load; SOST was not detected by RNAseq (data not shown). Pathway enrichment analysis of mechanically regulated genes using Enrichr (57–59) revealed associations with the bone remodelling/RANKL pathway (Biocarta 2016: 10/16 p = 7.20E-04 and Bioplanet 2019: 18/54 p = 0.048, respectively; **Supplementary Table 4**). The Elsevier pathway database showed that mechanically regulated genes were associated with aberant bone cell function in several diseases inlcuding ‘osteoclasts function in Osteopetrosis’ (10/19 p = 0.004), ‘osteoclast activation in Rheumatoid Arthritis’ (20/57 p = 0.022), ‘osteoclast activation in postmenopause’ (16/42 p = 0.017), ‘WNT signaling dysregulation in osteoblasts’ (7/15 p = 0.035), ‘osteoclast activation in Psoriatic Arthritis’ (16/47 p = 0.049), and ‘TNF and IL1B induce metalloproteinase synthesis in Osteoarthritis’ (14/38 p = 0.034) (**Supplementary Table 4**).

OPG and RANKL release was measured by ELISA. OPG release did not change over time in control cultures but was significantly increased by load at 24 hours (**Figure 3B**; 5.6-fold p=0.003; load at 24 hours vs load at 1 hour p=0.001). RANKL was below the level of detection in all cultures (data not shown).

#### 3.3.3 Inflammation

Pathophysiological loading of osteocytes in our 3D model regulated genes known to be involved in pathways linked to inflammation including genes associated with an acute and chronic inflammatory response (**Supplementary Table 3E**). Of the 371 genes listed in these terms, 69 genes were upregulated, and 32 genes downregulated by mechanical load (Padj <0.05). Many of these genes also belonged to GO terms related to NFkB signalling including ‘regulation of IkBk/NFkB signalling’ (GO:0043122; 70/224 Padj 0.002), ‘IkBk/NFkB signalling’ (GO:0007249; 23/62 Padj 0.007), ‘positive regulation of IkBk/NFkB signalling’ (GO:0043123; 51/171 Padj 0.018), and ‘NFkB binding’ (GO:0051959; 11/25 Padj 0.014) (**Supplementary Table 3**). Enrichr database analysis also revealed association with the NFkB pathway (Biocarta 2016: 12/21 p = 6.59E-04) (**Supplementary Table 4**).

**Figure 4:**
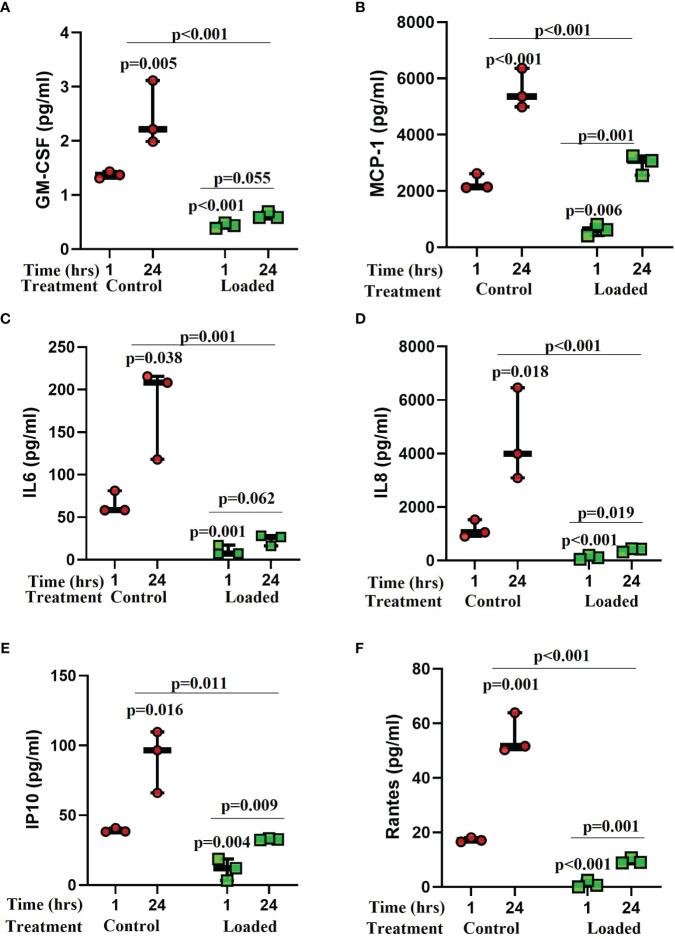
The effect of mechanical load on cytokine release from osteocytes grown in 3D collagen gels. Media was analysed for the release of cytokines 1 and 24 hours post load using a Luminex bead based multiplex assay (Merck Milliplex^®^ MAP human cytokine/chemokine magnetic bead panel kit). Samples were compared to control at 1 hour unless stated otherwise (n=3/treatment; GLM ANOVA with Tukey *post hoc* tests; IL-6 and GM-CSF logged data; IP-10 and RANTES 2-sample t-tests).

Several cytokines were detected in the control media from the osteocyte model (**Figure 4**) including GM-CSF, MCP-1, IL-6, IL-8, IP-10, RANTES, IL-10, IL-12p70, IL-5, and MIP1a. Levels of GM-CSF (1.8-fold vs 1-hr; p=0.005), MCP-1 (2.4-fold; p<0.001), IL-6 (2.7-fold; p=0.038), IL-8 (3.9-fold; p=0.018), IP-10 (2.3-fold; p=0.016), and RANTES (3.2-fold; p=0.001) increased over the 24-hrs in culture (**Figure 4**). Loading reduced the release of some cytokines after 1-hr and abolished the increase observed with time in culture (**Figure 4**). These included GM-CSF (1-hr vs control 1-hr: 3-fold, p<0.001; 24-hr vs control 24-hr: 3.9-fold, p<0.001), MCP-1 (1-hr 3.7-fold, p=0.006; 24-hr 3.2-fold, p<0.001), IL-6 (1-hr 6.3-fold, p=0.001; 24-hr 7.6-fold, p=0.001), IL-8 (1-hr 9.6-fold, p<0.001; 24-hr 11.3-fold, p<0.001), IP-10 (1-hr 3.5-fold, p=0.004; 24-hr 2.8-fold, p=0.011), and RANTES (1-hr 16.7-fold, p<0.001; 24-hr 5.8-fold, p<0.001). IL-10, IL-12p70, IL-5, and MIP1a were expressed but levels not elevated with time in culture or affected by load (data not shown).

### 3.4 IL-6 stimulation of osteocytes regulates readouts of osteoarthritis

Since IL-6 has been reported to be elevated in OA (31) and after knee injury significantly contributing to baseline KOOS and change in KOOS over 3 months (32), we stimulated our model with concentrations of IL-6 and its soluble receptor reported in synovial fluid from patients with OA (31) or following injury (32).

#### 3.4.1 Pain and the glutamate signalling pathway

**Figure 5:**
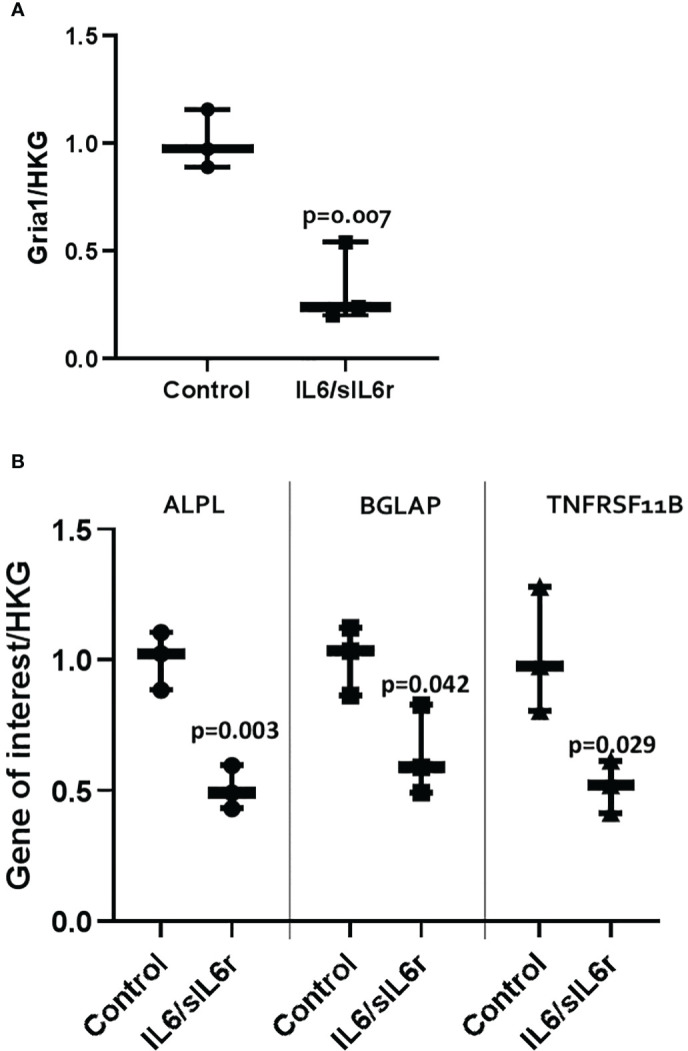
The effect of IL-6/sIL-6r on the expression of markers of **(A)** pain and **(B)** bone in osteocytes grown in 3D collagen gels. Gene expression was assessed at 24 hours post IL-6 (5ng/ml) and sIL-6r (40ng/ml) treatment and normalised to the geomean of two housekeeping genes (HKG), EEF and RPL13A. Samples were compared to control at 24 hours (One way ANOVA with Tukey *post hoc* tests; n=3).

IL-6/sIL-6r treatment downregulated GRIA1 (3-fold, p=0.007) mRNA expression (**Figure 5A**). SLClA1 and SLC1A3 were expressed but levels did not change with IL-6 treatment (data not shown). Glutamate release into the media increased with time in culture in control (p=0.005) and IL-6/sIL-6r (p=0.006) treated cultures but there was no effect of IL-6/sIL-6r treatment on glutamate release (**Supplementary Figure 4**).

#### 3.4.2 Bone remodelling

IL-6/sIL-6r treatment downregulated ALPL (2-fold; p=0.003), BGLAP (1.6-fold; p=0.042), TNFRSF11B (2-fold; p=0.029) mRNA expression (**Figure 5B**). Levels of Col1A1 did not change (data not shown). OPG release into the media increased with time in culture in control (p<0.001) and IL6/sIL6r (p<0.001) treated cultures but there was no effect of IL-6/sIL-6r treatment on OPG release (**Supplementary Figure 4**).

#### 3.4.3 Treatment of osteocytes with IL6/sIL6r increases the release of inflammatory mediators

**Figure 6:**
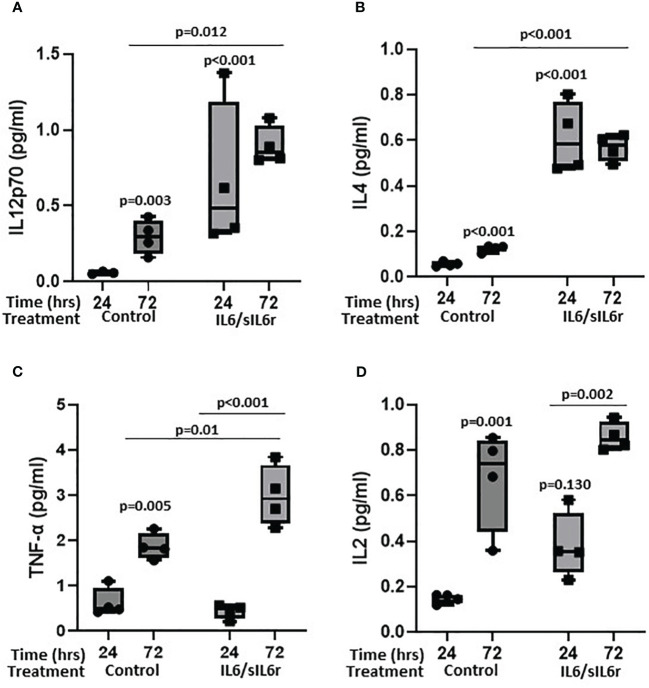
The effect of sIL-6r/IL-6 treatment on cytokine release from osteocytes grown in 3D collagen gels. Media was analysed for the release of **(A)** IL12p70, **(B)** IL4, **(C)** TNF-alpha, and **(D)** IL2 24 and 72 hours post treatment with IL-6 (5ng/ml) and sIL-6r (40ng/ml) using a multiplex ECL kit (Meso Scale Discovery). Samples were compared to control at 24 hour unless stated otherwise (n=3–4/treatment; GLM ANOVA with Tukey *post hoc* tests).

Several cytokines detected in control media increased over the 72 hours of culture including IL-12p70 (5-fold; p=0.003; **Figure 6A**), IL-4 (2.2-fold; p<0.001; **Figure 6B**), TNF-α (3-fold; p=0.005; **Figure 6C**), IL-2 (4.5-fold; p=0.001; **Figure 6D**), IFN-g (not expressed at 24-hrs but present at 72 hours; **Supplementary Figure 5A**), IL-10 (3-fold; p<0.001; **Supplementary Figure 5B**), IL-13 (2.2-fold; p=0.002; **Supplementary Figure 5C**), IL-1β (2-fold; p=0.055; **Supplementary Figure 5D**), and IL-8 (1.9-fold; p=0.017; **Supplementary Figure 5E**). Treatment of cells with IL-6/sIL-6r increased the amount of IL-12p70 (24-hrs 11.3-fold, p<0.001; 72-hrs 3-fold; p=0.012), IL-4 (24-hrs 12-fold, p<0.001; 72-hrs 4.6-fold, p<0.001), TNF-α (72-hr 1.6-fold p=0.01), IL-2 (24-hrs 2.3-fold, p=0.131), and IFN-g (72-hrs 2.4-fold, p=0.044) released into the media compared to untreated controls. IL-10, IL-13, IL-1β, and IL-8 levels were not changed by IL-6 treatment (**Supplementary Figures 5B–E**).

## 4 Discussion

### 4.1 A 3D model of human osteocyte like cells

**Figure 7:**
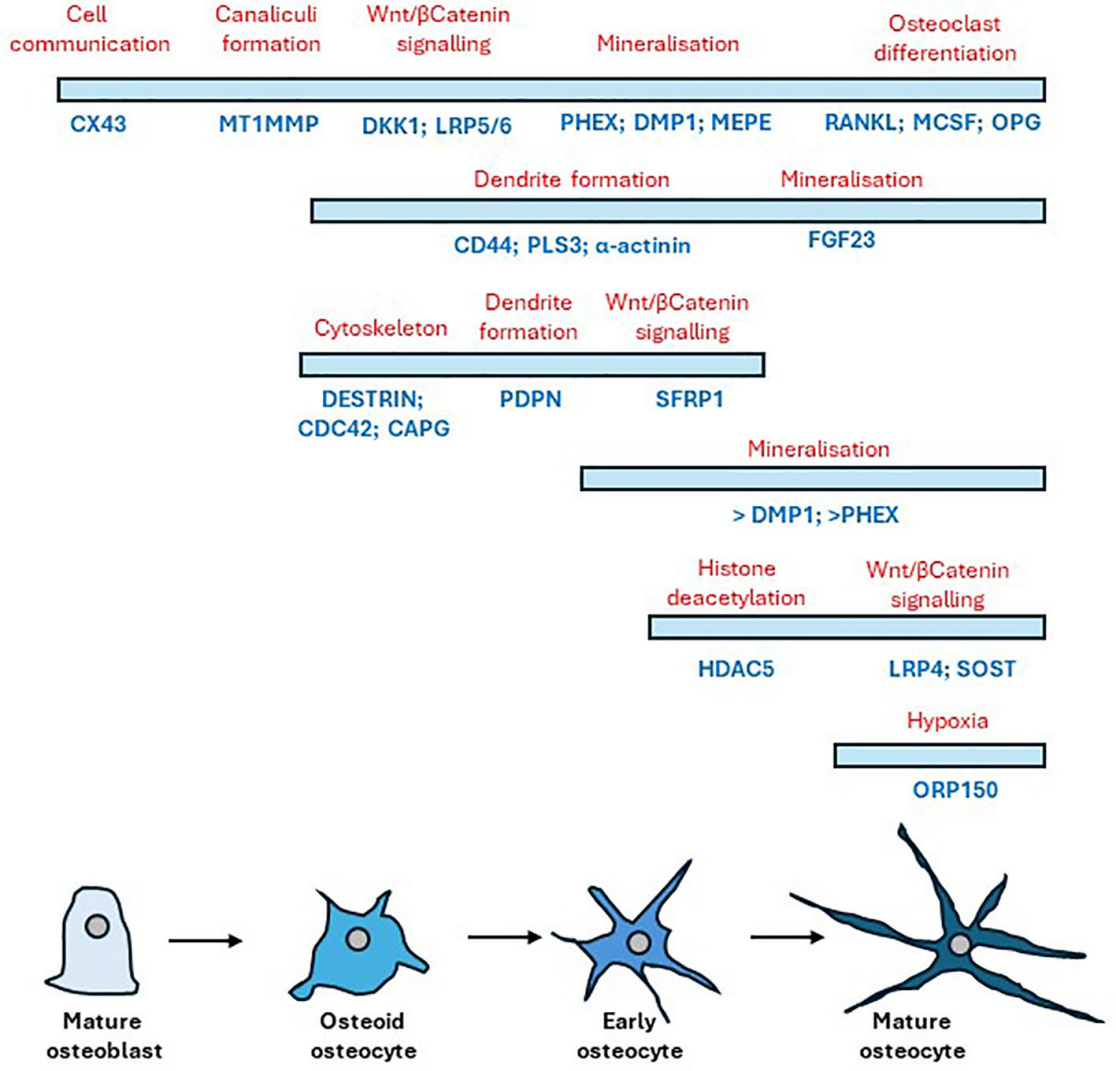
Timeline of gene expression changes that occur following the differentiation of mature osteoblasts to mature osteocytes. Bars indicate cell stage (blue) showing the relative temporal expression of various osteogenic markers underneath (dark blue) during the transition from osteoblast to osteocyte with their respective protein role above (red). Adapted from (72) and (10).

Y201 cells embedded in 3D collagen gels for 7 days displayed appropriated dendritic morphology (68), and expressed the mediator of osteocyte mechano-responses, sclerostin (69). SOST is not expressed in the early stages of differentiation of the osteoblast lineage, but levels increase as the osteocyte matures and become surrounded by mineralized bone (70). In addition, several genes were expressed that have been identified as being involved in the development and maturation of osteocytes [reviewed in (71–73)]. These included PDPN and CD44, markers of early osteocyte differentiation and required by osteocytes to initiate proper dendrite formation (74, 75), and a number of cytoskeletal proteins involved in actin dynamics such as PLS3, which encodes for an actin bundling protein required for osteocyte cytoskeleton organization (76), destrin, CAPG, and CDC42 (77). GJA1 (CX43) which is required for osteocyte communication (78), and MMP14, which is involved in canaliculi formation (72, 79) were also expressed along with DKK1, PHEX and FGF23 which play important roles in osteocyte development (72). BGLAP, COL1A1, TNFRSF11B, TNFSF11, PHOSPHO1, HDAC5, CYP19A1, RUNX2, DLX5, ATF4 and AP1 were also present representing genes that encode for proteins involved in osteocyte maturation and the mineralisation process [reviewed in (71)] (80). The genes involved in osteocyte development and maturation (5, 10, 70, 72, 74–79, 81–84) and found to be present in our dataset are summarized in **Figure 7**. Of the 1004 known human protein coding genes reported to reflect the *in vivo* osteocyte transcriptome signature (60), 93% were expressed in our model indicating that the cell phenotype and differentiation status is a good representation of osteocyte-like cells. The 7% of osteocyte signature genes not expressed in our RNAseq dataset included genes associated with the nervous system, the skeleton, angiogenesis, and cell function (**Supplementary Table 2C**). However, these may have been below the limit of detection since SOST was detected by RT-qPCR suggesting a reduced sensitivity in the RNAseq technology.

### 4.2 Mechanical loading regulates readouts of osteoarthritis in our 3D model of osteocytes

Mechanical loading decreased SOST expression in all 3D osteocyte cultures consistent with its regulation *in vivo* (46, 85) and regulated 70 genes linked to known mechanical responses by GO term enrichment confirming the model’s expected response to mechanical load. In total, mechanical loading of osteocytes down regulated 3026 genes and upregulated 4538 genes. Functional enrichment analysis using gProfiler revealed these were involved in many important cellular processes. Of note was the down regulation of processes involved in the primary cilia, an important key player in osteocyte mechanosensing (86) and bone formation in response to mechanical forces [reviewed in (87, 88)]. Thirty eight percent of the previously reported osteocyte signature genes (60) were regulated by mechanical load in our 3D model consistent with the dogma that osteocytes are highly responsive to mechanical load (60). Load regulated several genes associated with skeletal diseases such as osteopetrosis, osteoporosis and osteoarthritis in keeping with the human orthologs associated with osteoarthritis and osteoporosis reported in the *in vivo* mouse osteocyte transcriptome (60). Of the 25 genes of the osteocyte transcriptome signature associated with a skeletal phenotype (60), 15 were expressed but not regulated in our model, 3 were upregulated (CADM1, KAZN, STARD13) and 3 downregulated (CC2D2A, LTBP1, PLS3) by mechanical load (**Supplementary Table 5A**). Interestingly, mutation in PLS3 which encodes for plastin-3 an actin bundling protein required for organisation of the cytoskeleton in osteocytes (76) results in X-linked osteoporosis (89). In addition, of the 211 genes enriched within the signature that are associated with a skeletal phenotype in the Mouse Genome Informatics database (MGI) (60), our model expressed 108 genes that were not regulated by load, 50 that were upregulated, and 37 that were downregulated (**Supplementary Table 5B**). Bone marrow lesions (BMLs) occur in areas of bone remodelling and are associated with subchondral bone microdamage and correlate with pain in OA (67, 90, 91). Their presence and location are associated with altered joint loading as occurs in joint malalignment and absence/regression of BMLs occurs following reduction of focal contact stress across the joint (92). Joint pain resolution is associated with diminished BMLs. Comparison of our mechanosome data to the 78 DEGS identified as BML hub (93) revealed 56 genes to be expressed in our dataset, with 8 down regulated and 24 upregulated by load (**Supplementary Table 5C**). Additional components of BMLs such as COL12A1 (4-fold) and COL16A1 (1.7-fold), present in BMLs (66) which correlate to OA pain (67), were upregulated by load.

To determine whether mechanical load activated readouts classically associated with the structural and symptomatic changes in osteoarthritis, we performed gene ontology searches for pain, bone remodelling and inflammation and pathway enrichment analysis using Enrichr.

#### 4.2.1 Pain

Mechanical loading of osteocytes in our 3D model regulated 26% of the 253 genes known to be involved in gene ontology pathways linked to OA pain (**Supplementary Table 3C**). However, an important mediator of pain in human and animal osteoarthritis, Nerve Growth Factor (NGF), although expressed in all our osteocyte cultures, was not regulated by load (Padj = 0.067). Expression of NGF has previously been reported to be upregulated in osteoblasts by physiological mechanical forces although it was not detected in osteocytes (94). In addition, several of the proposed mediators of OA pain such as neuropeptide Y (NPY), and substance P were not expressed in our model. However, Neuropeptide Y 1 receptor (NPY1R) was expressed and mechanically downregulated in our osteocyte model, consistent with the mouse osteocyte transcriptome (60) but we did not detect NPY itself, also reported in osteocyte transcriptome. NPY is released by osteocytes, is important in balancing adipocyte and osteoblast differentiation and mediates its effects via receptors, NPY1R and NPY2R which localise to pain centres in the nervous system (95). NPY influences nociceptive signalling in neuropathic and inflammatory pain (96). It is an important regulator of bone homeostasis and its expression is increased in osteoarthritic synovium and the concentration in synovial fluid from osteoarthritic patients positively correlates with pain scores, and is increased in late stages of OA (97). Since osteocyte NPY acts through NPY1R to suppress osteogenesis and promote adipogenesis of bone marrow stem cells, the effect of the mechanically induced 70% reduction in NPY1R expression that we observed requires further study. We and others have previously shown that glutamate receptors are involved in neural responses to inflammatory pain and that glutamate receptor antagonists alleviate pain and degeneration in animal models (13, 22). AMPA (GRIA1/3/4), kainate (GRIK1/2/4/5) and NMDA (GRIN2A-D and 3A) ionotropic glutamate receptor subunits mRNAs were expressed in our osteocyte model. Of these GRIN2D and GRIK2 were identified in the osteocyte signature and involved with IDSWG3 maturation and GRIA3, GRIK5, GRIN2A and 3A in the osteocyte transcriptome (60). Kainate (GRIK2, 2-fold) and AMPA (GRIA3, 0.4-fold; GRIA4 20-fold) receptor subunits were mechanically regulated in osteocytes. This, along with reports of *in vivo* osteocyte expression of glutamate receptor proteins (AMPAR2 (13, 22); GRIN1, GRIA1/4 (14)), indicates a potential for mechanically-regulated glutamatergic signalling in osteocytes. Glutamate transporters (SLC1A1–3/EAAT1–3) were expressed by osteocytes consistent with previous reports *in vivo* (EAATs 1 and 2; SLC1A1 and 3 (60). Mechanical loading upregulated osteocyte SLC1A1/EAAT3 mRNA expression 3-fold but the mechanically-induced down regulation of SLC1A3/EAAT1 observed in osteocytes *in vivo* (16) was not recapitulated in this model. Upregulation of EAAT3 may explain why osteocytic glutamate release over 24 hours was reduced by mechanical loading. Increased concentrations of glutamate present in joint fluids in osteoarthritis, after joint injury, and at onset of joint inflammation (13, 22), are associated with pain and joint pathology (reviewed in (98)). The mechanically induced control of extracellular glutamate concentrations and regulation of glutamate receptor and transporter expression implicates osteocytes in the regulation of osteoarthritic pain and pathology although the effect of glutamate receptor activation in osteocytes is unknown.

#### 4.2.2 Bone remodelling

Mechanical loading of osteocytes in our 3D model regulated 40% (23% upregulated; 16% downregulated) of the 217 genes known to be involved in gene ontology pathways linked to bone remodelling (**Supplementary Table 3C**).

The nearly 6-fold increase in OPG protein release after loading, without detectable changes in RANKL protein expression reveals a potential mechanically-induced inhibition of osteoclastogenesis and bone resorption consistent with the role of osteocytes in regulating bone remodelling in response to their mechanical environment (99). Mechanically regulated genes reflected pathways associated with RANKL signalling and abnormal bone cell function in a range of skeletal diseases, involving osteoclast activation and dysregulation of WNT signalling in osteoblasts. Interestingly similar pathways were identified as those that differed in the osteocyte transcriptome with age and sex (60).

#### 4.2.3 Inflammation

Pathophysiological loading of osteocytes in our 3D model expressed 371 genes involved in acute and chronic inflammation with 27% of these genes being mechanically regulated (19% upregulated, 8% downregulated) (**Supplementary Table 3D**). NFkB signalling was particularly activated by mechanical load consistent with osteocytic loading playing a role in activating important proinflammatory and apoptotic pathways. In addition to the gene changes, several cytokine proteins were released by our osteocyte model, with mechanical loading reducing expression of both pro-resorptive (GM-CSF, IL-6, and RANTES) as well as anti-inflammatory (MCP-1, IL-8, IP-10) proteins. This indicates that osteocytes can directly link mechanical loading to inflammation potentially mediating pathological processes in mechanically driven diseases such as osteoarthritis.

### 4.3 IL-6 regulate readouts of osteoarthritis in our 3D model of osteocytes

IL-6 and its soluble receptor were used at concentrations reported in human synovial fluids from patients with OA (32) or following knee injury (31) to stimulate inflammation in our 3D osteocyte model. Although IL6/sIL6r downregulated the AMPA glutamate receptor mRNA, GRIA1, treatment with IL-6 had limited effects on the glutamate signalling pathway and did not regulate expression of either glutamate transporters or glutamate release. Conversely, IL-6 treatment did affect markers of bone remodelling, halving OPG expression and reducing indicators of bone formation (BGLAP) and mineralisation (ALPL) indicative of increased resorption and reduced osteogenesis. Treatment with IL-6/sIL-6r resulted in the release of both pro- and anti-inflammatory cytokines causing a >10-fold increase in IL-12p70 and IL-4 and approximately doubling TNF-α and IFN-γ protein release by osteocytes in our model all of which have been implicated in OA [reviewed in (100)] (30, 32),]. IL-4 and IL12 have been shown to be anti-osteoclastogenic and exert anti-resorptive effects on bone (101, 102). These data show that osteocytes in our 3D model respond to an inflammatory stimulus to modulate readouts of OA including markers of pain, bone remodelling and inflammation.

### 4.4 Links to human OA

We analysed our mechanosome dataset to determine whether genes that have been specifically identified as effector genes in OA patients were differentially expressed. A genome-wide association study (GWAS) meta-analysis across 13 international cohorts (826,690 individuals, 177,517 with osteoarthritis) identified 77 putative effector genes by analysis of functional genomics, fine-mapping, eQTL, and associations with animal and human musculoskeletal and neuronal phenotypes (65). Of the 77 genes with strong evidence as effector genes (score3+), 83% were expressed in our osteocyte model with nearly half mechanically regulated (17 upregulated; 15 downregulated) (**Supplementary Table 6A**). The druggable genome database (reference (103) in (65)), revealed that twenty tier 1 (approved/clinical-phase drugs), five tier 2 (binding partners to approved drug targets) and twenty tier 3 (druggable pathways) were associated with these 77 putative causal genes. Of the 32 mechanically regulated genes in our model, identified as ‘effector genes’, 10 represented potential druggable targets (tier 1: TGFB1, TNC, CTSK, NOS3; tier 3A: GDF5, LTBP3, SERPINF1, NOG, LTBP1; tier 3B: MGP). A GWAS study by Tachmazidou et al. found 9 genes underlying monogenic forms of bone development diseases and ten likely early OA effector genes (104). Six genes underlying monogenic diseases were expressed in our dataset (2 upregulated by load) and 7/10 OA effector genes were present (2 upregulated and 2 down regulated by load) (**Supplementary Table 6B**). In addition, a search of the OMIM® database (https://omim.org/) using the terms ‘osteoarthritis AND bone revealed 97 gene entries with a phenotype related to OA; 26 of the associated genes were regulated by load in our dataset, 16 up regulated and 10 down regulated and another further 38 genes were present but not regulated (**Supplementary Table 6C**). A recent study by Zhou et al. (105) also reported links between OA and mechanical responsive osteocyte genes including POSTN, NID2, and ASPN; all regulated by load in our dataset. Collectively, this data shows that mechanical loading of osteocytes in our model regulates the expression of several genes shown to be important in human osteoarthritis susceptibility and potential treatment.

### 4.5 Limitations

There are several limitations to our model. The type I collagen gel is not mineralised or organised as it would be *in vivo* and represents newly formed osteoid; with time this may become change and future studies could examine this. This means it does not exhibit the physical and chemical properties of bone and whilst the osteocyte-like cells appear to have good molecular and morphological phenotypes, these will not be completely the same as those *in vivo.* We have applied compressive strain based on measurements of the gel under loading, and mimicked strains observed in rodent bones (45). The mechanical environment experienced by the cells is not the same as it would be in a mineralised bone since the osteocytes are not located within lacunae with fluid flowing through them under load and the strain on the cell and the way the strain changes over the culture period is not defined. The cells are subjected to compressive, tensile stretch and fluid low all of which will influence the cell’s responses; further work, beyond the scope of the current study, could perform finite element modelling to help clarify this. Finally, the load was a single load of 3000 cycles that occurred over approximately 5 minutes. This could reflect joint trauma, or high strains caused by an episode of abnormal loading through the joint. The intention is to identify osteocyte derived mechanoresponsive signals that could contribute to disease processes in osteoarthritis, not to model the chronic disease and all joint tissues. We only used osteocytes in the current study; future work could co-culture osteoblasts and/or osteoclasts to mimic more closely the bone environment or other cell types to investigate tissue interactions. PCA analysis revealed a potential variation in differentiation status across cultures, with the gene expression profile of sample C2 varying somewhat from C3–5 and the L2 and L3 response to load varying from L4–6. This variation is likely due to subtle differences in cell density or distribution when embedding within the type I collagen, leading to a delay in cell-cell interactions, network connectivity and inhibition of cell division (10). Despite this, we have shown that our cells express an osteocyte phenotype using several approaches including RT-qPCR expression of osteocyte markers, high homology to the osteocyte signature, which are genes enriched in osteocytes relative to bone marrow and other osteoblast lineage cells (60), and protein expression of the mechanosensing osteocyte protein, sclerostin; the cells would not express high levels of sclerostin. A timeline of gene expression changes that occur following the differentiation of mature osteoblasts to mature osteocytes is shown in **Figure 7** [adapted from (10, 72)].

## 5 Conclusion

We have developed a reproducible model of human osteocyte like cells that express >90% of the genes in the osteocyte transcriptome signature. Mechanical loading and inflammatory stimulation regulated many genes and proteins implicated in osteoarthritis symptoms of pain as well as inflammation and degeneration underlying disease progression. Nearly half of the genes classified as ‘effectors’ in GWAS were mechanically regulated in this model. This model reveals that osteocyte mechanobiology plays an important role in osteoarthritic pathology. The model will be useful in identifying new mechanisms underlying bone and joint pathologies and testing drugs targeting those mechanisms.

## Data availability statement

The data presented in this study are deposited in the Mendeley Data repository, DOI: 10.17632/5md5rnybcs.1.

## Author contributions

SG: Writing – review & editing, Writing – original draft, Visualization, Validation, Supervision, Resources, Project administration, Methodology, Investigation, Funding acquisition, Formal Analysis, Data curation, Conceptualization. BE: Writing – review & editing, Validation, Methodology, Investigation. CB: Writing – review & editing, Validation, Methodology, Investigation, Funding acquisition. RJ: Writing – review & editing, Validation, Software, Methodology, Investigation, Data curation. SE: Writing – review & editing, Validation, Software, Methodology, Investigation, Formal Analysis, Data curation. DM: Writing – review & editing, Writing – original draft, Validation, Supervision, Resources, Project administration, Methodology, Funding acquisition, Formal Analysis, Conceptualization.

## References

[1] Guilak F. Biomechanical factors in osteoarthritis. Best Pract Res Clin Rheumatol. (2011) 25:815–23. doi: 10.1016/j.berh.2011.11.013 PMC3266544 22265263

[2] Watt FE Corp N Kingsbury SR Frobell R Englund M Felson DT. Towards prevention of post-traumatic osteoarthritis: report from an international expert working group on considerations for the design and conduct of interventional studies following acute knee injury. Osteoarthritis Cartilage. (2019) 27:23–33. doi: 10.1016/j.joca.2018.08.001 30125638 PMC6323612

[3] Kim JR Yoo JJ Kim HA. Therapeutics in osteoarthritis based on an understanding of its molecular pathogenesis. Int J Mol Sci. (2018) 19:674. doi: 10.3390/ijms19030674 29495538 PMC5877535

[4] Findlay DM Atkins GJ. Osteoblast-chondrocyte interactions in osteoarthritis. Curr Osteoporos Rep. (2014) 12:127–34. doi: 10.1007/s11914-014-0192-5 PMC3933767 24458429

[5] Bonewald LF. The amazing osteocyte. J Bone Miner Res. (2011) 26:229–38. doi: 10.1002/jbmr.320 PMC3179345 21254230

[6] Asada N Katayama Y Sato M Minagawa K Wakahashi K Kawano H. Matrix-embedded osteocytes regulate mobilization of hematopoietic stem/progenitor cells. Cell Stem Cell. (2013) 12:737–47. doi: 10.1016/j.stem.2013.05.001 23746979

[7] Bergwitz C Juppner H. Regulation of phosphate homeostasis by PTH, vitamin D, and FGF23. Annu Rev Med. (2010) 61:91–104. doi: 10.1146/annurev.med.051308.111339 20059333 PMC4777331

[8] Sato M Asada N Kawano Y Wakahashi K Minagawa K Kawano H. Osteocytes regulate primary lymphoid organs and fat metabolism. Cell Metab. (2013) 18:749–58. doi: 10.1016/j.cmet.2013.09.014 24140021

[9] Repp F Kollmannsberger P Roschger A Kerschnitzki M Berzlanovich A Gruber GM. Spatial heterogeneity in the canalicular density of the osteocyte network in human osteons. Bone Rep. (2017) 6:101–8. doi: 10.1016/j.bonr.2017.03.001 PMC5369863 28377989

[10] Dallas SL Prideaux M Bonewald LF. The osteocyte: an endocrine cell more. Endocr Rev. (2013) 34:658–90. doi: 10.1210/er.2012-1026 PMC3785641 23612223

[11] Qin L Liu W Cao H Xiao G. Molecular mechanosensors in osteocytes. Bone Res. (2020) 8:23. doi: 10.1038/s41413-020-0099-y 32550039 PMC7280204

[12] Hunter DJ Guermazi A Roemer F Zhang Y Neogi T. Structural correlates of pain in joints with osteoarthritis. Osteoarthritis Cartilage. (2013) 21:1170–8. doi: 10.1016/j.joca.2013.05.017 23973127

[13] Bonnet CS Gilbert SJ Blain EJ Williams AS Mason DJ. AMPA/kainate glutamate receptor antagonists prevent posttraumatic osteoarthritis. JCI Insight. (2020) 5:e134055. doi: 10.1172/jci.insight.134055 32544091 PMC7406304

[14] Chenu C Serre CM Raynal C Burt-Pichat B Delmas PD. Glutamate receptors are expressed by bone cells and are involved in bone resorption. Bone. (1998) 22:295–9. doi: 10.1016/S8756-3282(97)00295-0 9556127

[15] Flood S Parri R Williams A Duance V Mason D. Modulation of interleukin-6 and matrix metalloproteinase 2 expression in human fibroblast-like synoviocytes by functional ionotropic glutamate receptors. Arthritis Rheumatol. (2007) 56:2523–34. doi: 10.1002/art.22829 17665433

[16] Mason DJ Suva LJ Genever PG Patton AJ Steuckle S Hillam RA. Mechanically regulated expression of a neural glutamate transporter in bone: a role for excitatory amino acids as osteotropic agents? Bone. (1997) 20:199–205. doi: 10.1016/S8756-3282(96)00386-9 9071469

[17] Alstergren P Ernberg M Nilsson M Hajati AK Sessle BJ Kopp S. Glutamate-induced temporomandibular joint pain in healthy individuals is partially mediated by peripheral NMDA receptors. J Orofac Pain. (2010) 24:172–80. doi: 10.11607/jofph.24.2.05 20401355

[18] McNearney T Speegle D Lawand N Lisse J Westlund KN. Excitatory amino acid profiles of synovial fluid from patients with arthritis. J Rheumatol. (2000) 27:739–45. PMC7894989 10743819

[19] McNearney T Baethge BA Cao S Alam R Lisse JR Westlund KN. Excitatory amino acids, TNF-alpha, and chemokine levels in synovial fluids of patients with active arthropathies. Clin Exp Immunol. (2004) 137:621–7. doi: 10.1111/j.1365-2249.2004.02563.x PMC1809131 15320917

[20] Lawand NB McNearney T Westlund KN. Amino acid release into the knee joint: key role in nociception and inflammation. Pain. (2000) 86:69–74. doi: 10.1016/S0304-3959(99)00311-5 10779662

[21] Lam FF Ng ES. Substance P and glutamate receptor antagonists improve the anti-arthritic actions of dexamethasone in rats. Br J Pharmacol. (2010) 159:958–69. doi: 10.1111/j.1476-5381.2009.00586.x PMC2829221 20128799

[22] Bonnet CS Williams AS Gilbert SJ Harvey AK Evans BA Mason DJ. AMPA/kainate glutamate receptors contribute to inflammation, degeneration and pain related behaviour in inflammatory stages of arthritis. Ann Rheum Dis. (2015) 74:242–51. doi: 10.1136/annrheumdis-2013-203670 PMC4283694 24130267

[23] Gilbert SJ Bonnet CS Stadnik P Duance VC Mason DJ Blain EJ. Inflammatory and degenerative phases resulting from anterior cruciate rupture in a non-invasive murine model of post-traumatic osteoarthritis. J Orthop Res. (2018) 36(8):2118–27. doi: 10.1002/jor.23872 PMC6120532 29453795

[24] Chen W Ma Y Ye H He Y Li X Li J. ERK1/2 is involved in cyclic compressive force-induced IL-6 secretion in MLO-Y4 cells. Biochem Biophys Res Commun. (2010) 401:339–43. doi: 10.1016/j.bbrc.2010.09.044 20849821

[25] Sanchez C Gabay O Salvat C Henrotin YE Berenbaum F. Mechanical loading highly increases IL-6 production and decreases OPG expression by osteoblasts. Osteoarthritis Cartilage. (2009) 17:473–81. doi: 10.1016/j.joca.2008.09.007 18974013

[26] Bakker AD Kulkarni RN Klein-Nulend J Lems WF. IL-6 alters osteocyte signaling toward osteoblasts but not osteoclasts. J Dent Res. (2014) 93:394–9. doi: 10.1177/0022034514522485 24492932

[27] De Benedetti F Rucci N Del Fattore A Peruzzi B Paro R Longo M. Impaired skeletal development in interleukin-6-transgenic mice: a model for the impact of chronic inflammation on the growing skeletal system. Arthritis Rheumatol. (2006) 54:3551–63. doi: 10.1002/art.22175 17075861

[28] Rufo A Del Fattore A Capulli M Carvello F De Pasquale L Ferrari S. Mechanisms inducing low bone density in Duchenne muscular dystrophy in mice and humans. J Bone Miner Res. (2011) 26:1891–903. doi: 10.1002/jbmr.410 PMC3150693 21509823

[29] Feng W Liu H Luo T Liu D Du J Sun J. Combination of IL-6 and sIL-6R differentially regulate varying levels of RANKL-induced osteoclastogenesis through NF-kappaB, ERK and JNK signaling pathways. Sci Rep. (2017) 7:41411. doi: 10.1038/srep41411 28128332 PMC5269740

[30] Nees TA Rosshirt N Zhang JA Reiner T Sorbi R Tripel E. Synovial cytokines significantly correlate with osteoarthritis-related knee pain and disability: inflammatory mediators of potential clinical relevance. J Clin Med. (2019) 8:1343. doi: 10.3390/jcm8091343 31470613 PMC6780543

[31] Kotake S Sato K Kim KJ Takahashi N Udagawa N Nakamura I. Interleukin-6 and soluble interleukin-6 receptors in the synovial fluids from rheumatoid arthritis patients are responsible for osteoclast-like cell formation. J Bone Miner Res. (1996) 11:88–95. doi: 10.1002/jbmr.5650110113 8770701

[32] Watt FE Paterson E Freidin A Kenny M Judge A Saklatvala J. Acute molecular changes in synovial fluid following human knee injury: association with early clinical outcomes. Arthritis Rheumatol. (2016) 68:2129–40. doi: 10.1002/art.39677 PMC5006850 26991527

[33] Livshits G Zhai G Hart DJ Kato BS Wang H Williams FM. Interleukin-6 is a significant predictor of radiographic knee osteoarthritis: The Chingford Study. Arthritis Rheumatol. (2009) 60:2037–45. doi: 10.1002/art.24598 PMC2841820 19565477

[34] Blumenfeld O Williams FM Valdes A Hart DJ Malkin I Spector TD. Association of interleukin-6 gene polymorphisms with hand osteoarthritis and hand osteoporosis. Cytokine. (2014) 69:94–101. doi: 10.1016/j.cyto.2014.05.012 25022967

[35] Singh T Newman AB. Inflammatory markers in population studies of aging. Ageing Res Rev. (2011) 10:319–29. doi: 10.1016/j.arr.2010.11.002 PMC3098911 21145432

[36] Spector TD Hart DJ Nandra D Doyle DV Mackillop N Gallimore JR. Low-level increases in serum C-reactive protein are present in early osteoarthritis of the knee and predict progressive disease. Arthritis Rheumatol. (1997) 40:723–7. doi: 10.1002/art.1780400419 9125256

[37] Ryu JH Yang S Shin Y Rhee J Chun CH Chun JS. Interleukin-6 plays an essential role in hypoxia-inducible factor 2alpha-induced experimental osteoarthritic cartilage destruction in mice. Arthritis Rheumatol. (2011) 63:2732–43. doi: 10.1002/art.30451 21590680

[38] Wu X Cao L Li F Ma C Liu G Wang Q. Interleukin-6 from subchondral bone mesenchymal stem cells contributes to the pathological phenotypes of experimental osteoarthritis. Am J Transl Res. (2018) 10:1143–54. PMC5934573 29736207

[39] Johnson CI Argyle DJ Clements DN. *In vitro* models for the study of osteoarthritis. Vet J. (2016) 209:40–9. doi: 10.1016/j.tvjl.2015.07.011 26831151

[40] Samvelyan HJ Hughes D Stevens C Staines KA. Models of osteoarthritis: relevance and new insights. Calcif Tissue Int. (2021) 109:243–56. doi: 10.1007/s00223-020-00670-x PMC8403120 32062692

[41] Vazquez M Evans BA Riccardi D Evans SL Ralphs JR Dillingham CM. A new method to investigate how mechanical loading of osteocytes controls osteoblasts. Front endocrinology. (2014) 5:208.doi: 10.3389/fendo.2014.00208 PMC4260042 25538684

[42] James S Fox J Afsari F Lee J Clough S Knight C. Multiparameter analysis of human bone marrow stromal cells identifies distinct immunomodulatory and differentiation-competent subtypes. Stem Cell Rep. (2015) 4:1004–15. doi: 10.1016/j.stemcr.2015.05.005 PMC4471830 26070611

[43] Kim J Adachi T. Cell-fate decision of mesenchymal stem cells toward osteocyte differentiation is committed by spheroid culture. Sci Rep. (2021) 11:13204. doi: 10.1038/s41598-021-92607-z 34168224 PMC8225633

[44] Galarza Torre A Shaw JE Wood A Gilbert HTJ Dobre O Genever P. An immortalised mesenchymal stem cell line maintains mechano-responsive behaviour and can be used as a reporter of substrate stiffness. Sci Rep. (2018) 8:8981. doi: 10.1038/s41598-018-27346-9 29895825 PMC5997644

[45] Hillam RA Skerry TM. Inhibition of bone resorption and stimulation of formation by mechanical loading of the modeling rat ulna in vivo. J Bone Miner Res. (1995) 10:683–9. doi: 10.1002/jbmr.5650100503 7639102

[46] Robling AG Niziolek PJ Baldridge LA Condon KW Allen MR Alam I. Mechanical stimulation of bone in *vivo* reduces osteocyte expression of Sost/sclerostin. J Biol Chem. (2008) 283:5866–75. doi: 10.1074/jbc.M705092200 18089564

[47] Mason DJ Hillam RA Skerry TM. Constitutive in *vivo* mRNA expression by osteocytes of beta-actin, osteocalcin, connexin-43, IGF-I, c-fos and c-jun, but not TNF-alpha nor tartrate-resistant acid phosphatase. J Bone Miner Res. (1996) 11:350–7. doi: 10.1002/jbmr.5650110308 8852945

[48] Rubin C Turner AS Bain S Mallinckrodt C McLeod K. Anabolism. Low mechanical signals strengthen long bones. Nature. (2001) 412:603–4. doi: 10.1038/35088122 11493908

[49] Robling AG Turner CH. Mechanical signaling for bone modeling and remodeling. Crit Rev eukaryotic Gene expression. (2009) 19:319–38. doi: 10.1615/CritRevEukarGeneExpr.v19.i4 PMC3743123 19817708

[50] Xie F Xiao P Chen D Xu L Zhang B. miRDeepFinder: a miRNA analysis tool for deep sequencing of plant small RNAs. Plant Mol Biol. (2012) 5. doi: 10.1007/s11103-012-9885-2 22290409

[51] Livak KJ Schmittgen TD. Analysis of relative gene expression data using real-time quantitative PCR and the 2(-Delta Delta C(T)) Method. Methods. (2001) 25:402–8. doi: 10.1006/meth.2001.1262 11846609

[52] Taylor S Wakem M Dijkman G Alsarraj M Nguyen M. A practical approach to RT-qPCR-Publishing data that conform to the MIQE guidelines. Methods. (2010) 50:S1–5. doi: 10.1016/j.ymeth.2010.01.005 20215014

[53] Dobin A Gingeras TR. Mapping RNA-seq reads with STAR. Curr Protoc Bioinf. (2015) 51:11.14.1–19. doi: 10.1002/0471250953.bi1114s51 PMC4631051 26334920

[54] Love MI Huber W Anders S. Moderated estimation of fold change and dispersion for RNA-seq data with DESeq2. Genome Biol. (2014) 15:550. doi: 10.1186/s13059-014-0550-8 25516281 PMC4302049

[55] Benjamini Y Drai D Elmer G Kafkafi N Golani I. Controlling the false discovery rate in behavior genetics research. Behav Brain Res. (2001) 125:279–84. doi: 10.1016/S0166-4328(01)00297-2 11682119

[56] Reimand J Kull M Peterson H Hansen J Vilo J. g:Profiler–a web-based toolset for functional profiling of gene lists from large-scale experiments. Nucleic Acids Res. (2007) 35:W193–200. doi: 10.1093/nar/gkm226 PMC1933153 17478515

[57] Chen EY Tan CM Kou Y Duan Q Wang Z Meirelles GV. Enrichr: interactive and collaborative HTML5 gene list enrichment analysis tool. BMC Bioinf. (2013) 14:128. doi: 10.1186/1471-2105-14-128 PMC3637064 23586463

[58] Xie Z Bailey A Kuleshov MV Clarke DJB Evangelista JE Jenkins SL. Gene set knowledge discovery with enrichr. Curr Protoc. (2021) 1:e90. doi: 10.1002/cpz1.90 33780170 PMC8152575

[59] Kuleshov MV Jones MR Rouillard AD Fernandez NF Duan Q Wang Z. Enrichr: a comprehensive gene set enrichment analysis web server 2016 update. Nucleic Acids Res. (2016) 44:W90–7. doi: 10.1093/nar/gkw377 PMC4987924 27141961

[60] Youlten SE Kemp JP Logan JG Ghirardello EJ Sergio CM Dack MRG. Osteocyte transcriptome mapping identifies a molecular landscape controlling skeletal homeostasis and susceptibility to skeletal disease. Nat Commun. (2021) 12:2444. doi: 10.1038/s41467-021-22517-1 33953184 PMC8100170

[61] Carbon S Ireland A Mungall CJ Shu S Marshall B Lewis S. AmiGO: online access to ontology and annotation data. Bioinformatics. (2009) 25:288–9. doi: 10.1093/bioinformatics/btn615 PMC2639003 19033274

[62] Ashburner M Ball CA Blake JA Botstein D Butler H Cherry JM. Gene ontology: tool for the unification of biology. Gene Ontology Consortium. Nat Genet. (2000) 25:25–9. doi: 10.1038/75556 PMC3037419 10802651

[63] Gene Ontology C Aleksander SA Balhoff J Carbon S Cherry JM Drabkin HJ. The gene ontology knowledgebase in 2023. Genetics. (2023) 224:25–9. doi: 10.1038/75556 PMC10158837 36866529

[64] St John HC Bishop KA Meyer MB Benkusky NA Leng N Kendziorski C. The osteoblast to osteocyte transition: epigenetic changes and response to the vitamin D3 hormone. Mol Endocrinol. (2014) 28:1150–65. 10.1210/me.2014-1091 PMC5414828 24877565

[65] Boer CG Hatzikotoulas K Southam L Stefansdottir L Zhang Y Coutinho de Almeida R. Deciphering osteoarthritis genetics across 826,690 individuals from 9 populations. Cell. (2021) 184:4784–818 e17. doi: 10.1136/annrheumdis-2017-211396 34450027 PMC8459317

[66] Kuttapitiya A Assi L Laing K Hing C Mitchell P Whitley G. Microarray analysis of bone marrow lesions in osteoarthritis demonstrates upregulation of genes implicated in osteochondral turnover, neurogenesis and inflammation. Ann Rheum Dis. (2017) 76:1764–73. doi: 10.1136/annrheumdis-2017-211396 PMC5629942 28705915

[67] Felson DT Chaisson CE Hill CL Totterman SM Gale ME Skinner KM. The association of bone marrow lesions with pain in knee osteoarthritis. Ann Intern Med. (2001) 134:541–9. doi: 10.7326/0003-4819-134-7-200104030-00007 11281736

[68] Palumbo C Palazzini S Zaffe D Marotti G. Osteocyte differentiation in the tibia of newborn rabbit: an ultrastructural study of the formation of cytoplasmic processes. Acta Anat (Basel). (1990) 137:350–8. doi: 10.1159/000146907 2368590

[69] Tu X Rhee Y Condon KW Bivi N Allen MR Dwyer D. Sost downregulation and local Wnt signaling are required for the osteogenic response to mechanical loading. Bone. (2012) 50:209–17. doi: 10.1016/j.bone.2011.10.025 PMC3246572 22075208

[70] Dallas SL Bonewald LF. Dynamics of the transition from osteoblast to osteocyte. Ann New York Acad Sci. (2010) 1192:437–43. doi: 10.1111/j.1749-6632.2009.05246.x PMC2981593 20392270

[71] Delgado-Calle J Bellido T. The osteocyte as a signaling cell. Physiol Rev. (2022) 102:379–410. doi: 10.1152/physrev.00043.2020 34337974 PMC8858675

[72] Plotkin LI Bellido T. Osteocytic signalling pathways as therapeutic targets for bone fragility. Nat Rev Endocrinol. (2016) 12:593–605. doi: 10.1038/nrendo.2016.71 27230951 PMC6124897

[73] Dallas SL Prideaux M Bonewald LF. The osteocyte: an endocrine cell … and more. Endocrine Rev. (2013) 34:658–90. doi: 10.1210/er.2012-1026 PMC3785641 23612223

[74] Zhang K Barragan-Adjemian C Ye L Kotha S Dallas M Lu Y. E11/gp38 selective expression in osteocytes: regulation by mechanical strain and role in dendrite elongation. Mol Cell Biol. (2006) 26:4539–52. doi: 10.1128/MCB.02120-05 PMC1489126 16738320

[75] Hughes DE Salter DM Simpson R. CD44 expression in human bone: a novel marker of osteocytic differentiation. J Bone Miner Res. (1994) 9:39–44. doi: 10.1002/jbmr.5650090106 7512306

[76] Kamioka H Sugawara Y Honjo T Yamashiro T Takano-Yamamoto T. Terminal differentiation of osteoblasts to osteocytes is accompanied by dramatic changes in the distribution of actin-binding proteins. J Bone Miner Res. (2004) 19:471–8. doi: 10.1359/JBMR.040128 15040836

[77] Guo D Keightley A Guthrie J Veno PA Harris SE Bonewald LF. Identification of osteocyte-selective proteins. Proteomics. (2010) 10:3688–98. doi: 10.1002/pmic.201000306 PMC3517134 20845334

[78] Plotkin LI Bellido T. Beyond gap junctions: Connexin43 and bone cell signaling. Bone. (2013) 52:157–66. doi: 10.1016/j.bone.2012.09.030 PMC3513515 23041511

[79] Plotkin LI. Connexin 43 and bone: not just a gap junction protein. Actualizaciones En Osteologia. (2011) 7:79–90. doi: 10.1016/j.bone.2015.07.035 22679450 PMC3367377

[80] Javaheri B Carriero A Staines KA Chang YM Houston DA Oldknow KJ. Phospho1 deficiency transiently modifies bone architecture yet produces consistent modification in osteocyte differentiation and vascular porosity with ageing. Bone. (2015) 81:277–91. doi: 10.1016/j.bone.2015.07.035 PMC4652607 26232374

[81] Paic F Igwe JC Nori R Kronenberg MS Franceschetti T Harrington P. Identification of differentially expressed genes between osteoblasts and osteocytes. Bone. (2009) 45:682–92. doi: 10.1016/j.bone.2009.06.010 PMC2731004 19539797

[82] Sato T Verma S Andrade CDC Omeara M Campbell N Wang JS. A FAK/HDAC5 signaling axis controls osteocyte mechanotransduction. Nat Commun. (2020) 11:3282. doi: 10.1038/s41467-020-17099-3 32612176 PMC7329900

[83] Bernhardt A Weiser E Wolf S Vater C Gelinsky M. Primary human osteocyte networks in pure and modified collagen gels. Tissue Eng Part A. (2019) 25:1347–55. doi: 10.1089/ten.tea.2018.0338 30648477

[84] Boukhechba F Balaguer T Michiels JF Ackermann K Quincey D Bouler JM. Human primary osteocyte differentiation in a 3D culture system. J Bone Miner Res. (2009) 24:1927–35. doi: 10.1359/jbmr.090517 19419324

[85] Holguin N Brodt MD Silva MJ. Activation of wnt signaling by mechanical loading is impaired in the bone of old mice. J Bone Miner Res. (2016) 31:2215–26. doi: 10.1002/jbmr.2900 PMC5397287 27357062

[86] Temiyasathit S Jacobs CR. Osteocyte primary cilium and its role in bone mechanotransduction. Ann N Y Acad Sci. (2010) 1192:422–8. doi: 10.1111/j.1749-6632.2009.05243.x PMC3999479 20392268

[87] Chinipardaz Z Liu M Graves DT Yang S. Role of primary cilia in bone and cartilage. J Dent Res. (2022) 101:253–60. doi: 10.1177/00220345211046606 PMC8866845 34743626

[88] Yuan X Yang S. Primary cilia and intraflagellar transport proteins in bone and cartilage. J Dent Res. (2016) 95:1341–9. doi: 10.1177/0022034516652383 PMC5076748 27250654

[89] Makitie O Zillikens MC. Early-onset osteoporosis. Calcif Tissue Int. (2022) 110:546–61. doi: 10.1007/s00223-021-00885-6 PMC9013319 34236445

[90] Laslett LL Dore DA Quinn SJ Boon P Ryan E Winzenberg TM. Zoledronic acid reduces knee pain and bone marrow lesions over 1 year: a randomised controlled trial. Ann Rheum Dis. (2012) 71:1322–8. doi: 10.1136/annrheumdis-2011-200970 22355040

[91] Hunter DJ Guermazi A Lo GH Grainger AJ Conaghan PG Boudreau RM. Evolution of semi-quantitative whole joint assessment of knee OA: MOAKS (MRI Osteoarthritis Knee Score). Osteoarthritis Cartilage. (2011) 19:990–1002. doi: 10.1016/j.joca.2011.05.004 21645627 PMC4058435

[92] Callaghan MJ Parkes MJ Hutchinson CE Gait AD Forsythe LM Marjanovic EJ. A randomised trial of a brace for patellofemoral osteoarthritis targeting knee pain and bone marrow lesions. Ann Rheum Dis. (2015) 74:1164–70. doi: 10.1136/annrheumdis-2014-206376 PMC4771926 25596158

[93] Zeng MH Wang XS Chen TY Ruan GF Li J Xue S. Comprehensive analysis on subchondral bone marrow lesions of human osteoarthritis by integrating bulk and single-cell transcriptomes. BMC musculoskeletal Disord. (2023) 24:e3632–41. doi: 10.1186/s12891-023-06676-4 PMC10463447 37626330

[94] Tomlinson RE Li Z Li Z Minichiello L Riddle RC Venkatesan A. NGF-TrkA signaling in sensory nerves is required for skeletal adaptation to mechanical loads in mice. Proc Natl Acad Sci U S A. (2017) 114:E3632–E41. doi: 10.1073/pnas.1701054114 PMC5422802 28416686

[95] Lin ST Li YZ Sun XQ Chen QQ Huang SF Lin S. Update on the role of neuropeptide Y and other related factors in breast cancer and osteoporosis. Front endocrinology. (2021) 12:705499.doi: 10.3389/fendo.2021.705499 PMC8377469 34421823

[96] Tan CMJ Green P Tapoulal N Lewandowski AJ Leeson P Herring N. The role of neuropeptide Y in cardiovascular health and disease. Front Physiol. (2018) 9:1281.doi: 10.3389/fphys.2018.01281 30283345 PMC6157311

[97] Wang L Zhang L Pan H Peng S Lv M Lu WW. Levels of neuropeptide Y in synovial fluid relate to pain in patients with knee osteoarthritis. BMC Musculoskelet Disord. (2014) 15:319. doi: 10.1186/1471-2474-15-319 25262001 PMC4195915

[98] Wen ZH Chang YC Jean YH. Excitatory amino acid glutamate: role in peripheral nociceptive transduction and inflammation in experimental and clinical osteoarthritis. Osteoarthritis Cartilage. (2015) 23:2009–16. doi: 10.1016/j.joca.2015.03.017 26521747

[99] Robling AG Bonewald LF. The osteocyte: new insights. Annu Rev Physiol. (2020) 82:485–506. doi: 10.1146/annurev-physiol-021119-034332 32040934 PMC8274561

[100] Li J Zhang H Han Y Hu Y Geng Z Su J. Targeted and responsive biomaterials in osteoarthritis. Theranostics. (2023) 13:931–54. doi: 10.7150/thno.78639 PMC9925319 36793867

[101] Cheng J Liu J Shi Z Xu D Luo S Siegal GP. Interleukin-4 inhibits RANKL-induced NFATc1 expression via STAT6: a novel mechanism mediating its blockade of osteoclastogenesis. J Cell Biochem. (2011) 112:3385–92. doi: 10.1002/jcb.23269 PMC3580163 21751242

[102] Horwood NJ Elliott J Martin TJ Gillespie MT. IL-12 alone and in synergy with IL-18 inhibits osteoclast formation in vitro. J Immunol. (2001) 166:4915–21. doi: 10.4049/jimmunol.166.8.4915 11290769

[103] Finan C Gaulton A Kruger FA Lumbers RT Shah T Engmann J. The druggable genome and support for target identification and validation in drug development. Sci Transl Med. (2017) 9:eaag1166. doi: 10.1126/scitranslmed.aag1166 28356508 PMC6321762

[104] Tachmazidou I Hatzikotoulas K Southam L Esparza-Gordillo J Haberland V Zheng J. Identification of new therapeutic targets for osteoarthritis through genome-wide analyses of UK Biobank data. Nat Genet. (2019) 51:230–+. doi: 10.1038/s41588-018-0327-1 PMC6400267 30664745

[105] Zhou J He Z Cui J Liao X Cao H Shibata Y. Identification of mechanics-responsive osteocyte signature in osteoarthritis subchondral bone. Bone Joint Res. (2022) 11:362–70. doi: 10.1302/2046-3758.116.BJR-2021-0436.R1 PMC9233409 35678241

