# Supplementary figures and images for: Investigating mechanical and inflammatory pathological mechanisms in osteoarthritis using MSC-derived osteocyte-like cells in 3D
Source: Front Endocrinol (Lausanne). 2024 Aug 2;15:1359052. doi: 10.3389/fendo.2024.1359052 (PMC11328832; doi:10.3389/fendo.2024.1359052)

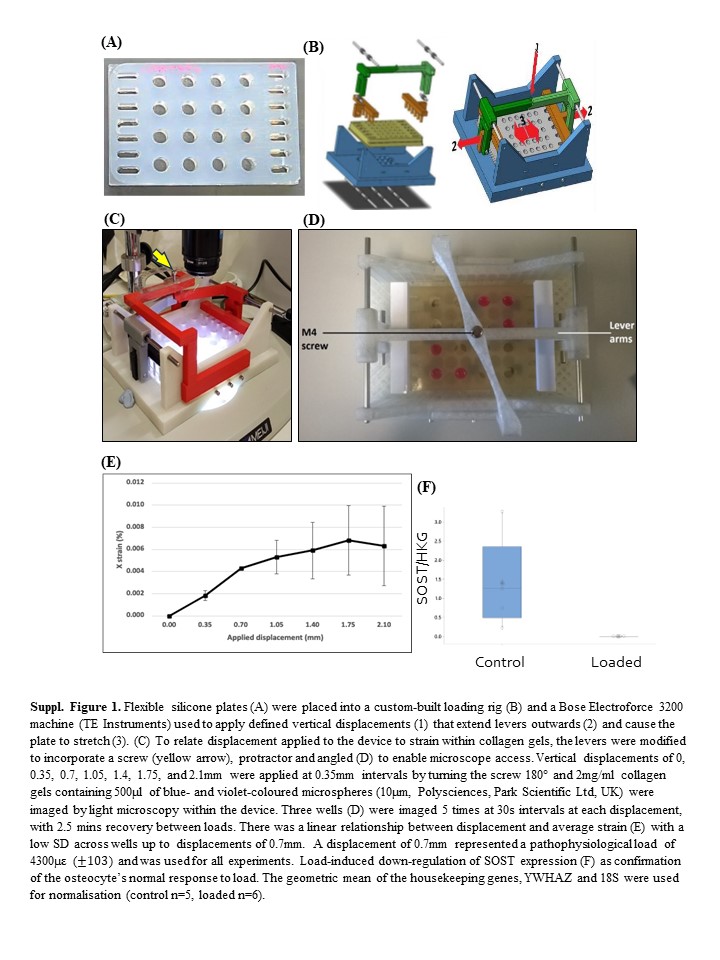

Supplement: Supplementary file 1 [file Image_1.jpeg]

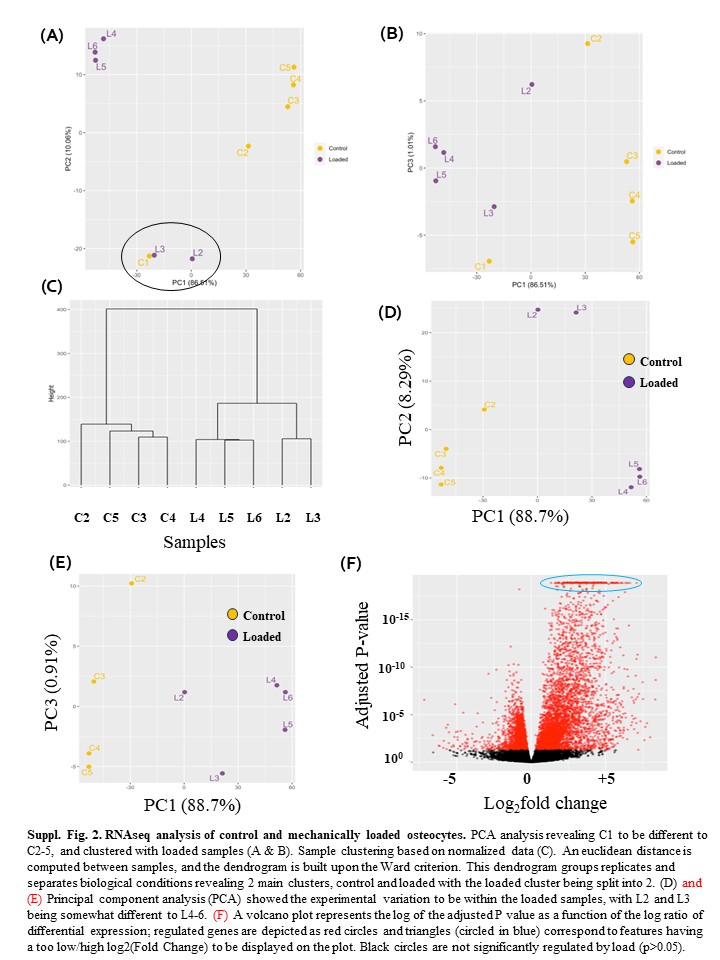

Supplement: Supplementary file 2 [file Image_2.jpeg]

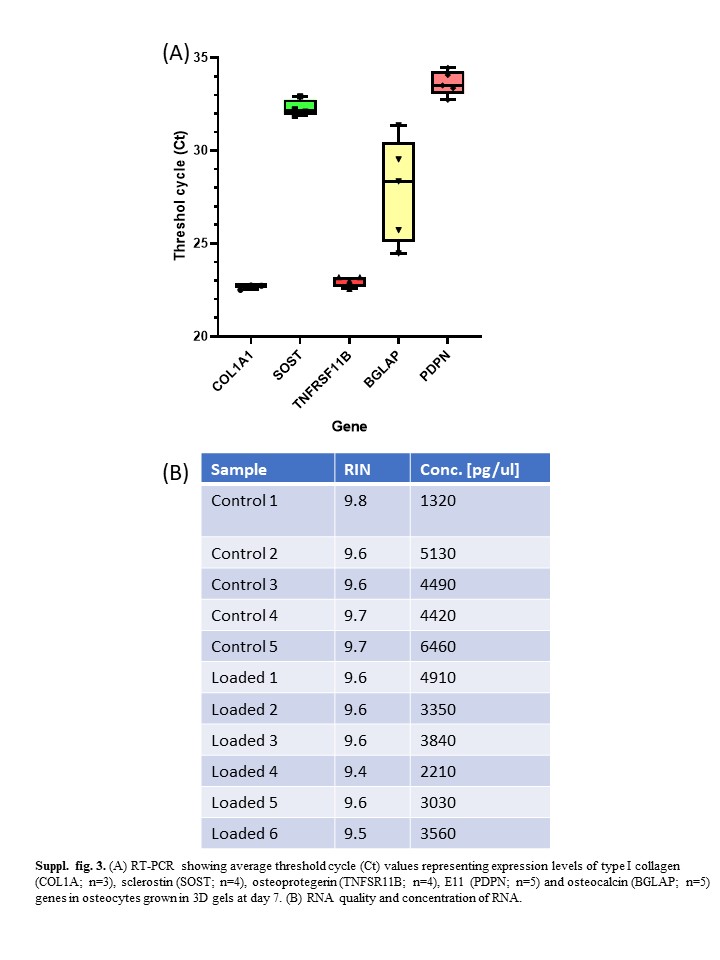

Supplement: Supplementary file 3 [file Image_3.jpeg]

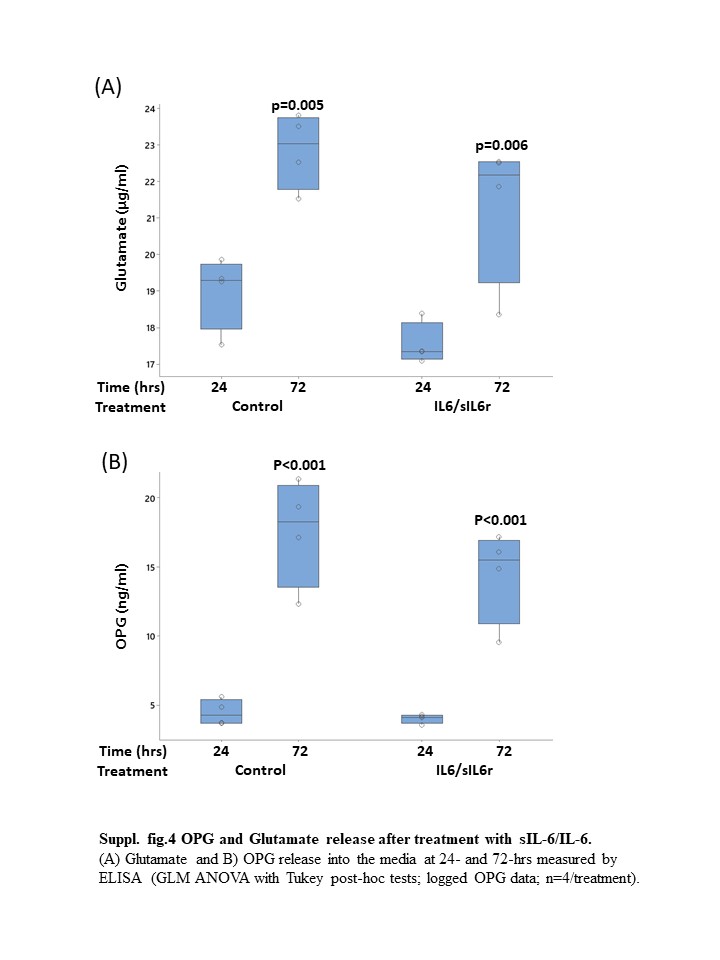

Supplement: Supplementary file 4 [file Image_4.jpeg]

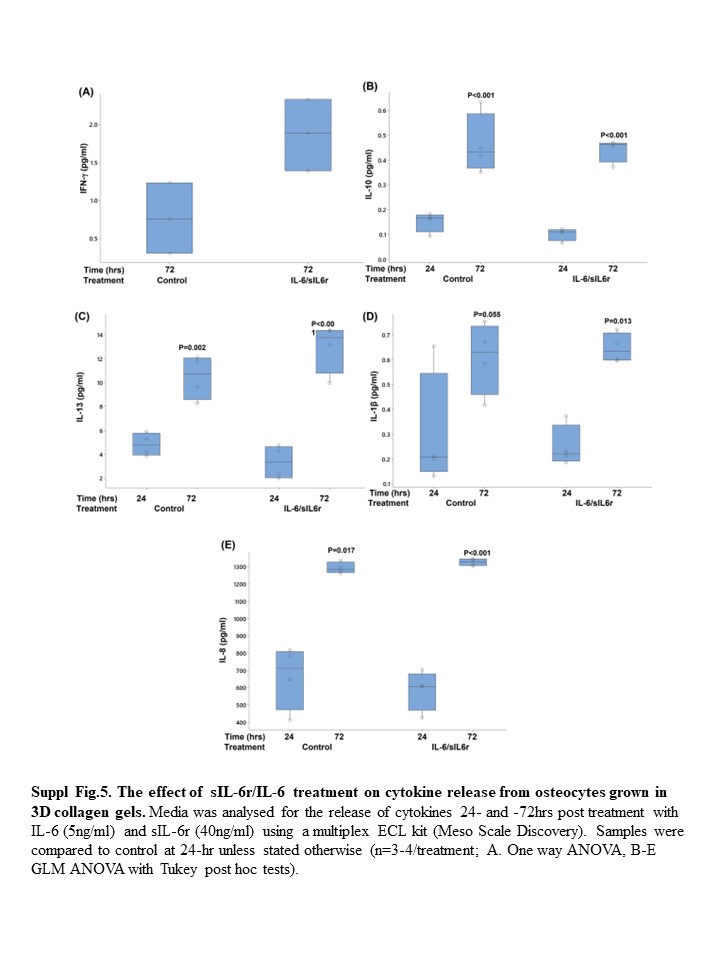

Supplement: Supplementary file 5 [file Image_5.jpeg]
