# Supplementary material for: Investigating mechanical and inflammatory pathological mechanisms in osteoarthritis using MSC-derived osteocyte-like cells in 3D
Source: Front Endocrinol (Lausanne). 2024 Aug 2;15:1359052. doi: 10.3389/fendo.2024.1359052 (PMC11328832; doi:10.3389/fendo.2024.1359052)
Supplement: Supplementary file 7 [file Table_1.docx]

Supplementary Material

# Supplementary Methods

# Silicone Plate Preparation

# Custom made 16 well silicone plates (1) were coated with 500μl rat tail type I collagen (Corning 0.15mg/ml in 0.2N glacial acetic acid) and left in the tissue culture hood for 12 hours to allow collagen to adhere to plate surfaces. Excess coating collagen was removed, and coated plates left to air dry in the tissue culture hood for 12 hours. Prepared plates were then ready for immediate use or were stored for up to one week at 4°C in sterile conditions.

# Supplementary Table 1

**Suppl. Table 1A: PCR Primer sequences**

| Gene | Primers (5’-3’) | Tm (°C) | Source /  Reference |
| --- | --- | --- | --- |
| YWHAZ | Fwd: AACTTGACATTGTGGACATC  Rev: AAAACTATTTGTGGGACAGC | 60 | NM_001135701 |
| 18S | Fwd: GCAATTATTCCCCATGAACG  Rev: GGCCTCACTAAACCATCCAA | 60 | NR_146119 (2) |
| EEF | Fwd: GTGGTGGTGGACTGTGTGTC  Rev: CGCTGGAAGGTCTGGTAGAG | 61 | NM_001961.4 |
| RPL13A | Fwd: GGATGGTGGTTCCTGCTG  Rev: TGGTACTTCCAGCCAACCTC | 60 | NM_012423.4 |
| ALPL | Fwd:GTACGAGCTGAACAGGAACAACG  Rev: CTTGGCTTTTCCTTCATGGTG | 62 | NM_000478.3 |
| SOST | Fwd: AGAGTACCCCGAGCCTCC  Rev: AGCTGTACTCGGACACGTCTTTG | 63 | NM_025237.3 (3) |
| BGLAP | Fwd: CTTTGTGTCCAAGCAGGAGG  Rev: CTGAAAGCCGATGTGGTCAG | 59 | NM_199173.6 |
| COL1A1 | Fwd: CTCCTGACGCACGGCC  Rev: CCGTTCTGTACGCAGGTGATT | 61 | NM_000088.4 (3) |
| GRIA1 | Fwd: ACACCCAAGGGGTCTGCCCT  Rev:CGTACCACCATTTGCTTTTCAGCTTGT | 66 | NM_001364167.2 |
| PDPN | Fwd: AAGATGGCTTGCCAGTAGTCA  Rev: ggcgagaaccttccagaaat | 60 | NM_010329.2 |

**Suppl. Table 1B: Luminex Cytokine panel**

| IFNa2 | IL-10 | IL-4 | IL-8 | MIP-1a | TNFa |
| --- | --- | --- | --- | --- | --- |
| Eotaxin | IL-12p40 | IL-5 | IL-15 | IL-17A | IL-1b |
| GM-CSF | IL-12p70 | IL-6 | IP-10 | IL-1Ra | IL-2 |
| IFNg | IL-13 | IL-7 | MCP-1 | RANTES |  |

**Suppl. Table 1C: Meso scale cytokine panel.** Samples were loaded in duplicate, average intra-assay CV (%) is shown.

| IL-2 (27.4) | IL-13 (21.0) | IL-10 (20.8) |
| --- | --- | --- |
| IFNg (45.1) | IL-4 (30.0) | IL-8 (1.8) |
| IL-1b (8.42) | TNFa (36.3) | IL-12p70 (39.2) |

**References**

1. Vazquez M, Evans BA, Riccardi D, Evans SL, Ralphs JR, Dillingham CM, et al. A new method to investigate how mechanical loading of osteocytes controls osteoblasts. Frontiers in endocrinology. 2014;5:208.

2. Frye SR, Yee A, Eskin SG, Guerra R, Cong X, McIntire LV. cDNA microarray analysis of endothelial cells subjected to cyclic mechanical strain: importance of motion control. Physiological genomics. 2005;21(1):124-30.

3. Boukhechba F, Balaguer T, Michiels JF, Ackermann K, Quincey D, Bouler JM, et al. Human Primary Osteocyte Differentiation in a 3D Culture System. J Bone Miner Res. 2009;24(11):1927-35.

# Supplementary Table 4 Gene ontology databases

| **Enrichr database** | **Pathway** | **No. of genes** | **p-value** | **Genes** | | | |
| --- | --- | --- | --- | --- | --- | --- | --- |
| Biocarta 2016 | Bone Remodeling Homo sapiens rankl Pathway | 10/16 | 7.20E-04 | IFNAR2  IKBKB  IRF9 | MAPK8  TRAF6  FOSL2 | IKBKG  RELA | NFKBIA  FOSL1 |
|  | NF-kB Signaling Pathway | 12/21 | 6.59E-04 | IKBKB  NFKBIA  IRAK1 | TRAF  FADD  IKBKG | MAP3K7  RELA  MYD88 | TNFRSF1A TAB1  TRADD |
| Bioplanet 2019 | RANKL signaling pathway | 18/54 | 0.048 | JUN  FHL2  NFATC1  PAPSS2  RELA | PTK2  RELB  IKBKB  NFKBIAMAPK8 | TRAF3  TRAF6  IKBKG  TAB1 | MAP3K7  SQSTM1  MAPK3 MAP2K7 |
| **Elsevier database** | **Pathway** | **No. of genes** | **p-value** | **Genes** | | | |
|  | Osteoclasts Function in Osteopetrosis | 10/19 | 0.0042 | CLCN7  IKBKB  TRAF6 | NFATC1  SLC4A2  TCIRG1 | OSTM1  NFKBIA | IKBKG  MAP3K7 |
|  | Osteoclast Activation in Rheumatoid Arthritis | 20/57 | 0.022 | JUN  MAP2K2  GSN  STAT1  STAT3 | NFATC1  ELK1  IKBKB  NFKBIA  MAPK8 | IRAK1  TRAF3  TRAF6  TAB1  RAF1 | MAP2K7MAP3K7BIRC2  MYD8  MAPK3 |
|  | Osteoclast Activation in Postmenopause | 16/42 | 0.0174 | JUN  GSN  MAP2K2  TRADD  MAPK3 | ELK1  TNFRSF1A  IKBKB | TAB1  MAP2K7  RAF1  MAP3K7 | NFATC1  TRAF6  NFKBIA  MAPK8 |
|  | WNT Signaling Dysregulation in Osteoblast | 7/15 | 0.0346 | GSK3B  PORCN | AXIN1  LRP5 | OSTM1  MYC | DVL1 |
|  | Osteoclast Activation in Psoriatic Arthritis | 16/47 | 0.0499 | GSN  STAT1  STAT3  NFATC1  RELA  RELB | IKBKG  TAB1  MAP3K7  BIRC2  MYD88 | IKBKB  NFKBIA  IRAK1  TRAF6  REL |  |
|  | TNF and IL1B Induce Metalloproteinase Synthesis in Osteoarthritis | 14/38 | 0.0338 | MAP2K3  JUN  MAP2K2  TRADD  RAF1 | TNFRSF1ANFKBIA  MAPK8  IRAK1  TRAF6 | MAPK3 MYD88 MAP2K7  MAP3K7 |  |
| **Gene ontology** | regulation of I-kappaB kinase/NF-kappaB signaling (GO:0043122) | 70/224 | 0.0018 | MAPKBP1SLC35B2  SLC44A2  ZFAND6  TRADD  PIDD1  PLEKHG5  F2R  TICAM1  JMJD8  TNFRSF1A  RELA  SHARPIN  IRAK1  TIFA  FLNA  TNFRSF10BFKBP1A | HSPB1  TNFAIP3IKBKB  TRIM8  PYCARD  PIM2  TRAF4  TRAF3  CARD19  TRAF6  CANT1  SLC20A1  NOD1  FADD  NDFIP  STAT1  PLK2  NEK6 | TFG  NUP62  CPNE1  TRIM27  IKBKG  MAP3K7  TRIM14  RBCK1  CACTIN  SQSTM1  OPTN  BIRC2  NLRX1  CC2D1A  TMEM9B  MYD88 PER1 | TMED4  TICAM2  RIPK2  SPHK2  PDPK1  TNFRSF19  BCL10  ATP2C1  CXXC5  PELI1 PPP5C  TNIP1  VAPA  TNIP2  REL  LTBR  MIB2 |
|  | IkBk/NFkB signalling (GO:0007249) | 23/62 | 0.007 | TICAM2  RIPK2  TRADD  NFKBIL1  BCL10  NFKBIA | TICAM1  RELA  TNFRSF1A  RELB  IKBKB | PRDX4  SHARPIN  NKIRAS2  TRAF6  TIFA  BIRC2 | BCL3  REL  IKBKG  RBCK1  TAB1  MAP3K7 |
|  | positive regulation of I-kappaB kinase/NF-kappaB signaling (GO:0043123) | 51/171 | 0.0178 | SLC35B2  SLC44A2  TRADD  IKBKB  TRIM8  TMED4  CANT1  SLC20A1  RELA  SHARPIN  TNFRSF10BFKBP1A  LTBR | TFG  NUP62  PIM2  TRIM27  IKBKG  MAP3K7  NOD1  BCL10  IRAK1  TIFA  FLNA  FADD  CC2D1A | TICAM2  RIPK2  PLEKHG5  F2R  TNFRSF19  TICAM1 ATP2C1  CXXC5  NDFIP1  NEK6  PLK2  MIB2  MYD88 | JMJD8  TNFRSF1A  TRAF6  PELI1  TRIM14  RBCK1  BIRC2  PPP5C  VAPA  TNIP2  REL  TMEM9B |
|  | NF-kappaB binding (GO:0051059) | 11/25 | 0.0145 | NFKBIA  GSK3B  HDAC2 | CDKN2A  FAF1  HDAC3 | BCL10  COMMD7  RELA | CPNE1  EP300 |
